# ProfileGrids as a new visual representation of large multiple sequence alignments: a case study of the RecA protein family

**DOI:** 10.1186/1471-2105-9-554

**Published:** 2008-12-22

**Authors:** Alberto I Roca, Albert E Almada, Aaron C Abajian

**Affiliations:** 1 Department of Molecular Biology and Biochemistry, 560 Steinhaus Hall, University of California, Irvine, California 92697-3900, USA

## Abstract

**Background:**

Multiple sequence alignments are a fundamental tool for the comparative analysis of proteins and nucleic acids. However, large data sets are no longer manageable for visualization and investigation using the traditional stacked sequence alignment representation.

**Results:**

We introduce ProfileGrids that represent a multiple sequence alignment as a matrix color-coded according to the residue frequency occurring at each column position. JProfileGrid is a Java application for computing and analyzing ProfileGrids. A dynamic interaction with the alignment information is achieved by changing the ProfileGrid color scheme, by extracting sequence subsets at selected residues of interest, and by relating alignment information to residue physical properties. Conserved family motifs can be identified by the overlay of similarity plot calculations on a ProfileGrid. Figures suitable for publication can be generated from the saved spreadsheet output of the colored matrices as well as by the export of conservation information for use in the PyMOL molecular visualization program.

We demonstrate the utility of ProfileGrids on 300 bacterial homologs of the RecA family – a universally conserved protein involved in DNA recombination and repair. Careful attention was paid to curating the collected RecA sequences since ProfileGrids allow the easy identification of rare residues in an alignment. We relate the RecA alignment sequence conservation to the following three topics: the recently identified DNA binding residues, the unexplored MAW motif, and a unique *Bacillus subtilis *RecA homolog sequence feature.

**Conclusion:**

ProfileGrids allow large protein families to be visualized more effectively than the traditional stacked sequence alignment form. This new graphical representation facilitates the determination of the sequence conservation at residue positions of interest, enables the examination of structural patterns by using residue physical properties, and permits the display of rare sequence features within the context of an entire alignment. JProfileGrid is free for non-commercial use and is available from . Furthermore, we present a curated RecA protein collection that is more diverse than previous data sets; and, therefore, this RecA ProfileGrid is a rich source of information for nanoanatomy analysis.

## Background

Comparative nanoanatomy and phylogenetic studies of macromolecules depend upon multiple sequence alignments (MSAs). However, the traditional stacked sequence representation of an alignment proves cumbersome for large numbers of homologs as is prevalent with the proliferation of genome sequences. Early MSA formatting programs facilitated analysis by emphasizing residues with boxes, colors, and shading [1-3]. However, these programs (and many subsequent different implementations) still represent a MSA as stacked sequences. Regular expressions, major components [4], and sequence logos [5] are solutions to compress the sequence alignment information of motifs into a consensus format as reviewed in 2005 [6]. In addition, a graphical view of MSA conservation can be achieved with an "overview" mode [7,8] or with plots of similarity values [9]. However, all of these representations do not convey the details of each character's frequency distribution at each homologous position in the entire alignment. Thus, potentially valuable information for the interpretation of macromolecular structure and function is lost. Clearly there is a need for a new visual representation paradigm for MSAs.

Here we introduce the JProfileGrid Java software for generating ProfileGrids – a new graphical, tabular representation of alignments. Historically, profiles scored by a distance matrix were used for database searches [10], although simple frequency profiles have been used to tabulate the amino acid content of linear motifs [11]. By contrast, ProfileGrids are color-coded tables of the residue frequency occurring at every homologous position across the entire length of an MSA. Therefore, all MSA information is represented especially at variable regions and of rare residues that may yield clues about function. Similar to ColorGrids [12], the frequency determines color shading; but, ProfileGrids are specific for MSAs. In particular, our JProfileGrid software enables a dynamic visualization of structural patterns by analyzing protein alignments with respect to amino acid physical properties. Notably, JProfileGrid provides a unique method for generating publishable figures of the entire sequence content of an alignment with many homologs. A ProfileGrid facilitates the inspection of large MSAs and, thus, solves the problem of text legibility of traditional MSAs [13]. Below we describe the features of the JProfileGrid software and demonstrate a ProfileGrid's usefulness by examining the bacterial RecA protein family that we introduce next.

The RecA protein is the premier genomic sentinel of *Escherichia coli *because of its crucial protective roles in both recombinational DNA repair [14] and the SOS response [15]. RecA homologs are present in all domains of life [16,17] and well distributed among bacteria [18-21]. As the vanguard of bacterial RecA homologs, the *E. coli *RecA protein (352 residues; [GenBank:AAC75741.1]) has been intensively studied starting with its discovery [22] and the subsequent sequencing of its gene [23,24]. Later, many RecA sequences became available as microbiologists cloned *recA *genes from different culturable bacteria to construct knockout derivatives [25]. Furthermore, the ubiquity of the RecA homolog made it a common marker for phylogenetic studies [20] using the most conserved parts of the RecA protein – the adjacent MAW and P-loop motifs. The precise function of the former is unknown [17], while the latter motif is the well-characterized ATP-binding site [26].

RecA MSAs have been analyzed from a structural perspective to understand RecA function [17,27]. For example, molecular genetics approaches have generated over 1400 *E. coli *RecA missense mutations [28]; and, the phenotypes are discussed within the context of the sequence conservation occurring at the mutation location. Furthermore, conserved residues often have functional roles such as ligand binding so such positions are targets for inspection when studying protein structure. The recent determination of a RecA-DNA cocrystal structure [29] with the first clear identification of a DNA binding site provides a new motivation for RecA MSA information.

As the number of RecA homologs has increased, however, the visualization and analysis of a MSA becomes unwieldy using the traditional stacked sequence representation. In fact, the last complete RecA MSAs available as published figures comes from the mid-1990's when there were only about 60 homologs [17,19,30]. More recently, no MSA figures were included in the data sets of 144 [20] and 113 [21] RecA homologs. Since there are more RecA sequences available now, this family makes an excellent case study for showing how ProfileGrids succinctly display the information content of a large MSA. The present work describes a curated data set of 300 RecA protein sequences from a larger diversity of bacterial species than of previously reported alignments. The breadth of this sequence collection creates a robust description of the conserved sequence motifs of the RecA protein family and, therefore, may, shed light on unexplored regions of this protein such as the aforementioned MAW motif.

## Implementation

JProfileGrid is a Java program that combines the tasks of examining amino acid frequencies across an entire MSA, identifying conserved motif regions, and comparing species-specific residues against a sequence family. Both a command-line and a graphical user interface are available with the latter allowing interactive ProfileGrid analysis. The program accepts protein and nucleic acid MSAs in either MSF or FASTA formats. The former is preferred because of the inclusion of sequence weight values in the MSF file header. The similarity plot calculations are based on the plotcon algorithm [9] with a modification that the values are normalized between 0 and 1. The program saves matrix output as a spreadsheet file using the JExcel API [31]. The color formatted ProfileGrid and the similarity values are stored in separate worksheets. A third worksheet identifies outlier characters (such as "X") in the MSA that the program flags for verification. JProfileGrid can also write PyMOL scripts [32] that identify the conserved regions of the MSA on a protein structure.

## Methods

### Sequence data set

RecA protein sequences were collected from the following databases: the National Center for Biotechnology Information GenBank database [33], The Institute for Genomic Research Comprehensive Microbial Resource [34], the DNA Data Bank of Japan [35], the European Molecular Biology Laboratory Sequence Database [36], and UniProt [37]. Keyword searches were used at the aforementioned database websites especially for annotated genomes where RecA orthologs had already been identified. In addition, sequence similarity searches were performed using the *E. coli *RecA homolog as the query sequence in BLASTp and TBLASTN searches [38] with default parameters. After manually verifying the presence of conserved RecA family motifs, we added the protein sequences from the keyword search results and significant BLAST search hits (E-value <10^-70^) to our previous collection of validated bacterial RecA orthologs [17]. Since we focused on fully sequenced homologs from known bacterial species, no explicit attempt was made to collect RecA homologs from environmental sequencing projects such as from the Sargasso Sea collection [39]. In a previous analysis of 64 RecA homologs, 12 sequences were found to contain errors [17,40,41]. Although some of those have not yet been updated in GenBank, we used the corrected versions in all cases. Finally, we limited the RecA data set to unique sequences for each bacterial species. Specifically, we eliminated redundant sequences from duplicate sequencing efforts (genome versus individual gene projects) and from strains of the same bacterial species (*E. coli *CFT073 versus K12). While these sequences do not appear in our RecA MSA and ProfileGrid, the redundant sequences serve to verify any rare residue observations that could be the result of errors. This underscores the curation that was performed of the individual sequences as described in more detail below.

### Alignment

The multiple sequence alignments were calculated using the DNASTAR MegAlign program [42] that implements the ClustalW algorithm [43]. Default parameters were used except that the gap penalty was increased to 30 to minimize the introduction of gaps. The resulting alignment was manually curated by visual inspection to optimize the position of small gaps. Weight values were assigned to each protein sequence using the ClustalX program [44] to remove any bias from similar sequences potentially overrepresented in the alignment. The MegAlign program was also used to identify alignment positions that were either invariant or chemically similar (Additional file 1) according to previously described amino acid classes [17].

### Data curation

In the genomic era, database web interfaces make it easy for the novice user to find and align many RecA sequences. However the quality of the sequence data sets and their subsequent alignment can not be taken for granted. Instead it is imperative that bioinformatic data be curated to enable researchers to be confident of the conclusions that they draw [45]. This can be particularly important in the conserved motifs of a protein sequence alignment. Below, we belabor this point as a caution about the interpretation of rare residues in MSAs.

Inspection of the MSA (Additional file 1) and ProfileGrid (Additional file 2) show that the family motifs are very well conserved among the 300 RecA homologs. However, there are exceptions where residues occur which do not follow the consensus patterns for the motifs. These rare residues are readily visible in ProfileGrid representations. Such rare amino acids may be interesting exceptions or just noise in the bioinformatic data. We paid particular attention to the MAW and P-loop motifs that are the most conserved parts of the RecA family. For example, a single serine is observed in the MAW motif at *E. coli *position 52 where 298 other RecA sequences have glycine at that position (Additional file 2). This is not considered a conservative substitution. By contrast, a single serine in the P-loop at position 73 could be a conservative substitution when compared to the 299 other threonine residues. Structure and function inferences drawn from exceptions to conserved motifs would be a waste of effort if such exceptions were based upon faulty data. We also note that phylogenetic analyses are greatly affected by sequence errors [46].

Problems in sequence data sets can result from experimental artifacts or data handling mistakes. These issues are diminishing in the genomic era, but anomalies still occur. As mentioned above, we have identified errors in *recA *gene sequences determined using traditional gel techniques [41]. More importantly, genome projects are introducing a new problem where the complete determination of an organism's DNA content yields sequences that may not be true chromosomal RecA orthologs. For example, the *Salmonella enterica *genome project [47] uncovered both plasmid encoded [GenBank:CAD09875.1] and chromosome encoded [GenBank:CAD05935.1] RecA proteins. Only the latter was included in the work presented here. In addition, JProfileGrid will flag outliers of one letter characters that do not represent the common amino acids or gap codes. For example, in the RecA protein alignment reported here, we unexpectedly identified "X" characters in two sequences [GenBank:CAD79373.1, GenBank:AAN06665.1].

Significantly, this point about data curation is not just a hypothetical cautionary comment. Attention [48] was drawn to the observation of a rare tyrosine residue in the *Proteus vulgaris *RecA protein [49] where the vast majority of RecA homologs have serine at *E. coli *position 70 (Additional file 2). However the discrepancy was resolved [41] when it was determined that the tyrosine observation was actually a simple typographical error in the publication figure. Compounding this problem, though, was a data handling error of the *P. vulgaris *[GenBank:CAB56804.1] and *Pectobacterium carotovorum *(formerly *Erwinia carotovora*) [GenBank:CAB56783.1] RecA protein sequences both determined by the same group [49]. The sequence database records for these homologs were apparently mixed together such that the sequences do not agree with the protein sequences reported in the reference publication. The corrected sequences are used in this work. Thus, we encourage users of ProfileGrids to be cautious of overinterpreting rare residues identified in motifs. Currently, the accurate biocuration of sequence and alignment data sets can only be achieved by slow, tedious, manual efforts by protein family experts [50].

## Results and Discussion

### JProfileGrid software

**Figure 1:**
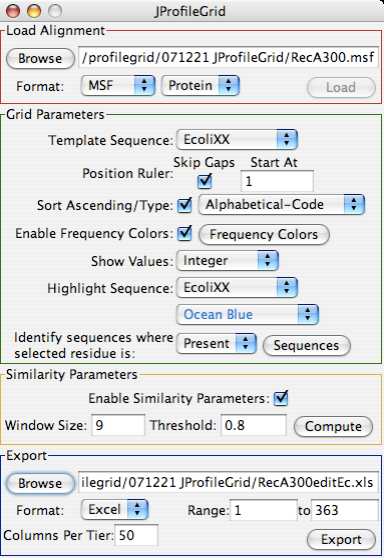
**A screen shot of the JProfileGrid parameter settings window**.

**Figure 2:**
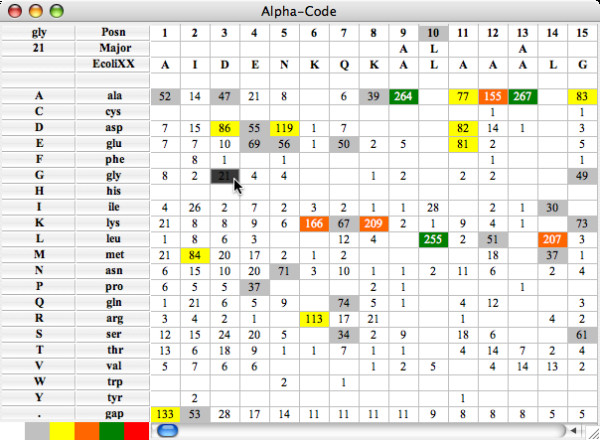
**The ProfileGrid viewer showing the RecA protein family results**. The first 3 rows of the ProfileGrid are a position ruler (Posn), a majority consensus (Major), and a template sequence (here of the *E. coli *RecA homolog). The remaining rows tabulate the frequency of the amino acid and gap characters at each position of the alignment. Cells are color shaded according to the frequency value (Figure 3). The top-left corner identifies the character and the frequency of the ProfileGrid cell currently selected by the cursor.

**Figure 3:**
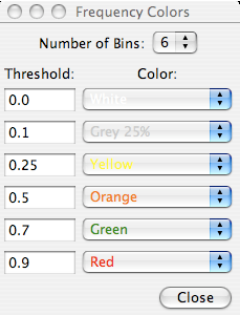
**The frequency settings determining a ProfileGrid cell color**.

The program is controlled from the parameter settings window (Figure 1) which is arranged from top-to-bottom for loading an alignment, customizing the appearance of a ProfileGrid, calculating the similarity plot values, and exporting the results. The ProfileGrid viewer (Figure 2) shows the results of the JProfileGrid calculation after opening the alignment file (here of the RecA family of 300 sequences). The first 3 rows are a position ruler, a majority consensus, and a template sequence (here of the *E. coli *RecA homolog). The next 21 rows tabulate the frequency of the amino acid and gap characters at the corresponding MSA column position. ProfileGrid cells are color shaded according to the residue frequency value (Figure 3) with the legend in the lower-left corner of the ProfileGrid viewer read from left to right as low to high conservation, respectively. The top-left corner identifies the character and the frequency of the ProfileGrid cell currently selected by the cursor. Note that each column total equals the number of sequences in the alignment. Since the ProfileGrid matrix needs only 21 residue rows to represent protein sequences, there is practically no limit to the number of homologs that can be visualized.

The parameter settings window (Figure 1) allows the user to change the template sequence, the position ruler numbering, the majority consensus sequence threshold cutoff (default 70%), and the residue sort order. By default, the template is the first sequence of the alignment; and, the amino acids are alphabetized by the one-letter code to facilitate looking up a residue of interest. JProfileGrid provides a menu of the following amino acid physical constants for analysis: age [51], flexibility [52], frequency among *E. coli *proteins [53], hydropathy [54], hydrophobicity [55], helix propensity [56], mutability [57,58], surface area [59], and volume [60]. Many more constants are available for those coding their own ProfileGrid implementations [61]. The "Frequency Colors" button opens a window listing the 6 default frequency color bins (Figure 3). A ProfileGrid cell is colored by the following bin that has the largest threshold value greater than or equal to a cell's residue frequency: <10% (white), ≥ 10% (gray), ≥ 25% (yellow), ≥ 50% (orange), ≥ 70% (green), and ≥ 90% (red). This color scheme was chosen to maximize the visual differences between bins for the inspection of ProfileGrids for patterns (see below). By contrast, a color ramp (*i.e.*, shades of one color) would not facilitate such analysis. However, the user is able to define their own frequency color scheme by choosing the number, size, and color of the bins. To assist the inspection of ProfileGrids, the frequency values can be hidden. This same menu allows the values to be reported as a percentage.

**Figure 4:**
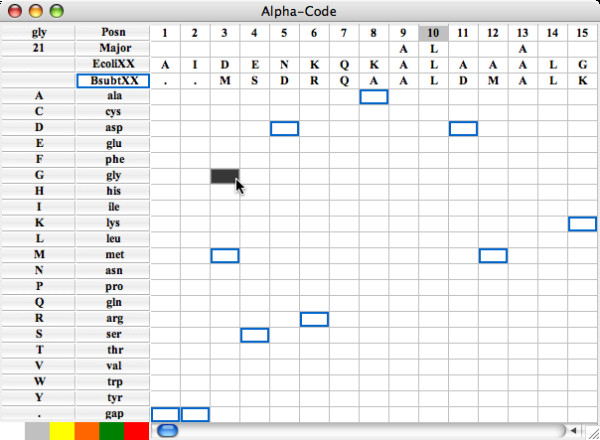
***B. subtilis *RecA highlight sequence example with frequency colors and values turned off**.

**Figure 5:**
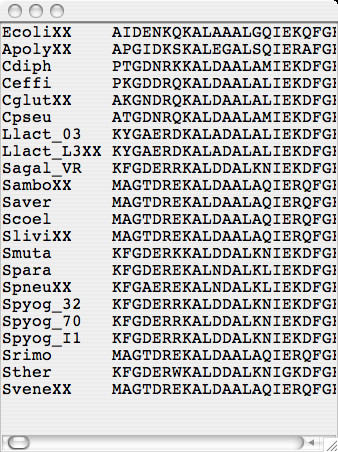
**The alignment viewer showing sequences from the currently selected ProfileGrid cell**.

Two features allow one to visualize other sequences of the ProfileGrid besides the template sequence. First, the highlight sequence option allows one to detect and to represent unique features of one sequence with respect to the entire information content of a MSA. Such a feature may indicate specialization with respect to function or activity. When the highlight menu is used to select a sequence different from the template sequence, then the highlight feature is turned on (Figure 4). Specifically, the highlight sequence will appear immediately below the template sequence in the ProfileGrid. Furthermore, a pairwise comparison is made such that the corresponding residue is boxed if the highlight sequence differs from the template sequence. The user may choose other colors besides the default blue selection. Note that in the highlight sequence figure, the cell value identification feature (top left corner) reports the current cell frequency even when the ProfileGrid colors and values are hidden. The second feature to visualize MSA sequences is the alignment viewer window (Figure 5) that displays a traditional alignment representation of sequences from the currently selected ProfileGrid cell. In this example, the 21 homologs that have glycine in the third column are shown. For comparison purposes, the first row in the alignment is the template sequence.

**Figure 6:**
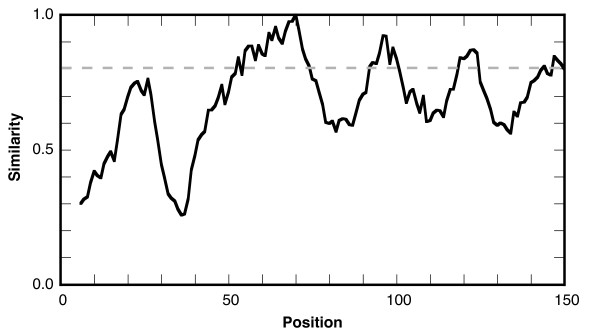
**Similarity plot of the RecA protein family**. Similarity values over the first 150 residues of the alignment were calculated using the BLOSUM62 scoring matrix and a window size of 9. A threshold value of 0.8 is indicated by the dashed line. A complete plot using a smaller RecA data set has been previously published [17].

**Figure 7:**
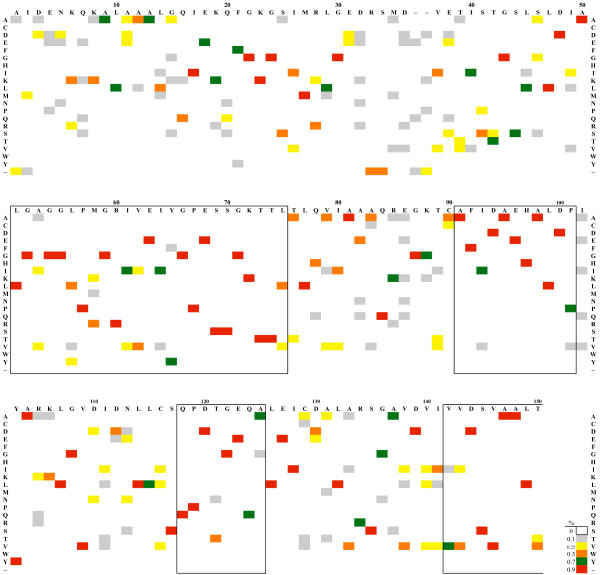
**ProfileGrid of 300 bacterial RecA protein sequences**. The first row is the *E. coli *RecA protein sequence. The ProfileGrid cells are colored according to the following bins: <10% (white), ≥10% (gray), ≥25% (yellow), ≥50% (orange), ≥70% (green), ≥90% (red). The boxed regions (potential motifs) were drawn by JProfileGrid from the similarity plot calculations using an 80% threshold cutoff. For visual clarity, only the first 150 residues of the alignment are shown; and, the frequency values are omitted. Additional File 2 is the entire RecA ProfileGrid including frequency values. This figure was generated from the JProfileGrid spreadsheet output.

JProfileGrid calculates similarity plot values (Figure 6) based on the plotcon algorithm [9]. A user-defined sliding window (default 5 residues) is used to calculate conservation across the MSA using the BLOSUM62 or EDNAFULL scoring matrices for proteins and nucleic acids, respectively. Weights for each sequence are taken from MSF input files to correct for overrepresented sequences. By contrast, calculations based upon FASTA files will not have such a correction. The similarity plot results can be visualized directly within a ProfileGrid. This is accomplished by a threshold cutoff value determining the endpoints of similarity boxes outlined in black in the ProfileGrid (Figure 7). These boxes emphasize conserved regions in the protein family. The similarity boxes also serve as landmarks when the ProfileGrid frequency cell colors are not shown.

**Figure 8:**
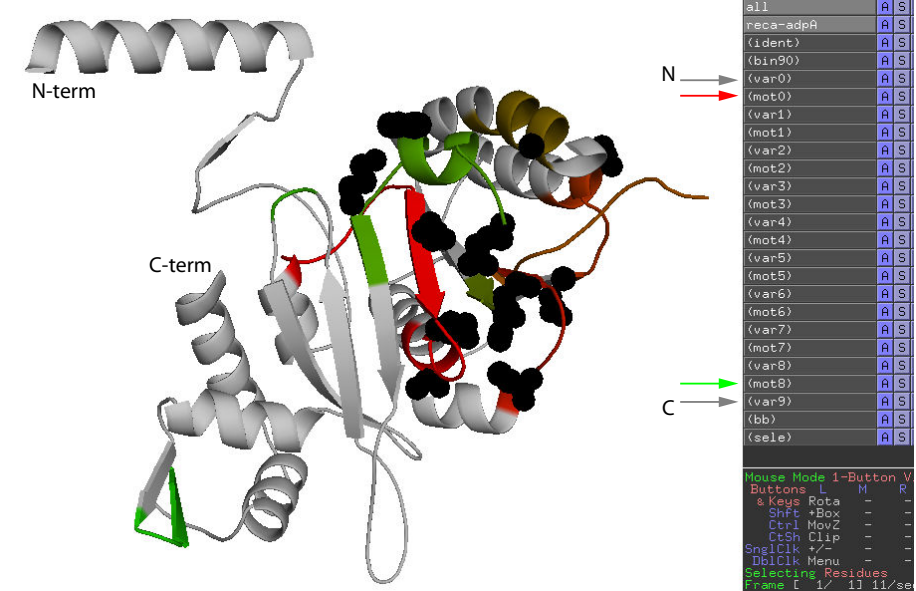
**Visualization of PyMOL script output**. JProfileGrid can write a ".pml" file that will define the following named selections based upon the ProfileGrid information: identical residues (black sidechains); conserved motifs ("mot#") colored from most amino terminal (red) to most carboxyl terminal (green); and connecting variable ("var#") regions (gray). These different selections are mapped on to the *E. coli *RecA crystal structure [PDB:2REB]. This orientation is defined as the anterior view of the RecA monomer anatomical position. Some of the named selections are indicated by arrows in this PyMOL screen shot.

JProfileGrid exports output in two formats. ProfileGrid figures for publication are made from a saved Excel spreadsheet file where the matrix appearance can be optimized such as the selection of the text font. The user can specify a subset range of MSA columns as well as the size of each ProfileGrid tier which in this example was set to 50 (Figure 7). A second output format is a script option for the PyMOL molecular visualization program (Figure 8) here showing the *E. coli *RecA crystal structure [62]. Residues that are completely conserved, *i.e.*, identical, in the MSA are saved as a PyMOL selection named "ident" in the script file. Residues that pass the highest threshold value in conservation (default bin of ≥90%) are saved as a selection named "bin90". Finally, the motifs and connecting variable regions are labeled numerically starting from the N-terminus.

### RecA family data set

We have analyzed a set of bacterial RecA homologs consisting of 300 near full-length protein sequences (Table 1). Approximately 280 of the sequences were full-length. The rest are missing short sequences at the termini. The number of unique bacterial species in the 300 sequence data set is 245. We included sequences from multiple strains of a single species whenever such sequences were unique. For example, five strains of *Streptococcus pyogenes *provided RecA sequences that differed at a small number (1 to 8) of residues. The sizes of the full-length sequences ranged from 318 (*Bacteroides fragilis*; GenBank: AAA22918.1) to 447 amino acids (*Tropheryma whipplei*; GenBank:AAO44708.1) with an average length of 354 ± 18. The degree of identity to the *E. coli *RecA protein sequence ranged from 37% (*Ureaplasma parvum*; GenBank:AAF30489.1) to 100% (*Shigella flexneri*; GenBank:AAP18040.1) with an average identity of 62% ± 10%. These calculations excluded the intein sequences found in the *Mycobacterium *RecA protein homologs [63].

**Table 1:** Bacterial RecA Homologs

**Phyla**	**1997**	**Current**	**Representative Species**
Actinobacteria	6	37	*Mycobacterium tuberculosis*
Aquificae	1	3	*Aquifex pyrophilus*
Bacteroidetes/Chlorobi	1	8	*Bacteroides fragilis*
Chlamydiae/Verrucomicrobia	1	8	*Chlamydia trachomatis*
Chloroflexi	0	2	*Dehalococcoides ethenogenes*
Cyanobacteria	3	12	*Anabaena variabilis*
Deinococcus-Thermus	3	5	*Deinococcus radiodurans*
Dictyoglomi	0	1	*Dictyoglomus thermophilum*
Fibrobacteres/Acidobacteria	0	2	*Fibrobacter succinogenes*
Firmicutes	8	73	*Bacillus subtilis*
Fusobacteria	0	2	*Fusobacterium nucleatum*
Nitrospirae	0	1	*Thermodesulfovibrio yellowstonii*
Planctomycetes	0	2	*Gemmata obscuriglobus*
Proteobacteria	(39)	(133)	
Alpha	11	30	*Rhodobacter capsulatus*
Beta	6	25	*Neisseria gonorrhoeae*
Delta/Epsilon	3	20	*Campylobacter jejuni*
Gamma	19	58	*Escherichia coli*
Spirochaetes	1	8	*Borrelia burgdorferi*
Thermodesulfobacteria	0	1	*Thermodesulfobacterium commune*
Thermotogae	1	2	*Thermotoga maritima*
		
Total	64	300	

Bacterial phylogenetic classification was taken from the NCBI Taxonomy database [33]. Column "1997" depicts the number of bacterial RecA homologs used in the multiple sequence alignment from a previous analysis [17]. The adjacent column shows the number of homologs used in the present work. The last column lists a representative species from the corresponding phyla.

The data sets from the mid-1990's [17,19,30] were biased toward RecA homologs from the Proteobacteria phyla (60% of sequences). In the current work, the purple bacteria represent only 44% of the sequences (Table 1). Furthermore, we now include homologs from several newly sequenced bacterial phyla including the Chlororflexi and the Fusobacteria. The diversity of the current data set permits a robust description of motifs of the RecA protein family. Additional file 1 shows a summary of the information from the RecA MSA.

### RecA family ProfileGrid applications

An alignment of 300 bacterial RecA homologs is graphically represented by a ProfileGrid (Figure 7). This visualization gives a succinct overview of MSA information especially when the frequency values are hidden to reduce clutter. The details of the residue frequency for all columns of the RecA MSA are found in Additional file 2. We used the sequence conservation denoted by the similarity boxes to define RecA motifs to serve as a nomenclature across the full length of the RecA protein family (see Additional files 1 and 2). The labeling (and subsequent analysis) of every part of the RecA protein is a fundamental technique adapted from traditional anatomy [64] and applied to macromolecules, *i.e.*, nanoanatomy.

The detailed RecA ProfileGrid information will allow researchers to examine conservation at RecA positions of interest. For example, a new suppressor mutation was recently [65] reported that ameliorates the effects of an impaired [KR]x[KR] motif [66]. The suppressor maps to *E. coli *RecA position 11 and is a change from alanine to valine which is a residue that is *not *observed among any of the 300 sequences in the MSA (Figure 2, Additional file 2). Since the current sequence data set is larger and more diverse than previous RecA homolog collections, one can have more confidence in the *lack *of an observed residue change.

The sequence conservation can also be related to RecA protein structure. For example, most of the 21 invariant residues (100% identity) are located on the monomer anterior side (Figure 8) that faces the central axis of the right-handed helical protein filament. The RecA filament interior is where the DNA strand exchange activity takes place. More specifically, a recent crystal structure of a RecA-DNA complex identifies residues involved in DNA binding [29]; but, the report did not discuss the sequence conservation of these amino acids. We observe that most of the positions involved in direct DNA contacts are almost completely conserved throughout bacterial RecA evolution (Table 2) as would be expected for ligand binding residues. However, there are some exceptions. In the *E. coli *RecA protein cocrystal structure, 164-met is involved in making DNA ribose contacts. Surprisingly, at this position methionine occurs in only 20% of the RecA homologs in the MSA. Instead valine is the more frequent (62%) residue found among bacterial RecA proteins. In addition, two residues involved in DNA base contacts (197-met and 199-ile) have potentially non-conservative substitutions with respect to charge (glutamate) or steric (valine) considerations, respectively. An *E. coli *RecA mutant 197-met to glu is defective for *in vivo *repair activities [67]. There are conflicting reports on whether a 199-ile to val RecA mutant is impaired for repair activity [67,68]. Parenthetically, we also checked these residue positions in MSAs of the distant RecA homologs such as eukaryotic Rad51/Dmc1, archaeal RadA, and viral UvsX proteins [17,69]. In contrast to the bacterial RecA MSA, only 211-gly and 212-gly are completely conserved among distant homologs while there is weak sequence similarity at positions 164, 176, 200, and 213. Models for the roles of the DNA-interacting positions should account for this sequence diversity.

**Table 2:** Conservation of DNA binding residues

**Residue**	**% Freq.**	**Other residues**
162-Ser	59	Ala 14%, Gln 12%
164-Met	20	Val 62%
165-Gly	99	
168-Ala	100	
169-Arg	99	
172-Ser	99	
176-Arg	99	
196-Arg	99	
197-Met	47	Glu 42%
198-Lys	98	
199-Ile	74	Val 25%
200-Gly	99	
207-Glu*	99	
208-Thr	90	
211-Gly	100	
212-Gly	99	
213-Asn	52	Arg 31%
226-Arg*	97	
243-Arg*	56	Lys 41%
245-Lys*	96	
280-Lys*	30	Glu 32%, Asp 16%
282-Lys*	19	Gly 36%, Asp 29%
286-Lys*	93	
302-Lys*	46	Arg 47%

The first column lists *E. coli *RecA residues directly involved in DNA binding and those residues proposed (*) to interact with DNA [29]. The "% Freq." column reports the percent frequency of the indicated amino acid among 300 RecA homologs. The last column shows the percent frequency of other residues at that position of the alignment. See text for a description of conservation at these positions among eukaryotic and archaeal RecA homologs.

### ProfileGrid structural pattern analysis of the MAW motif

When combined with different amino acid properties [61], ProfileGrids are a useful tool for visualizing structural patterns across the interspecies diversity of a protein family. We illustrate this on two adjacent motifs (MAW and P-loop) that comprise the most conserved part of RecA homologs of bacteria, eukaryotes, and archaea [17]. Of the two, only the function of the P-loop (the cofactor binding site) has been determined [26]. By contrast, little [17] is known about the MAW motif (residues 40–65). From the RecA crystal structures, the MAW motif (or "motif 1a"; see Additional file 1 for motif and variable names) consists of a loop, α-helix B, a tight turn, and ends with β-strand 1. This glycine-rich motif threads through the RecA hydrophobic core and interacts with motifs (1b, 4a, and 5b) that form part of the ATP binding site; but, the MAW region itself has not been shown to contact the cofactor ligand. The MAW motif also connects the P-loop to a hinge (variable 1) that undergoes a dramatic change in the transition from the inactive to active RecA conformation [29]. We note that aside from the protein termini, this hinge region is one of the least conserved parts of the RecA protein (Figure 6, Additional files 1 and 2).

**Figure 9:**
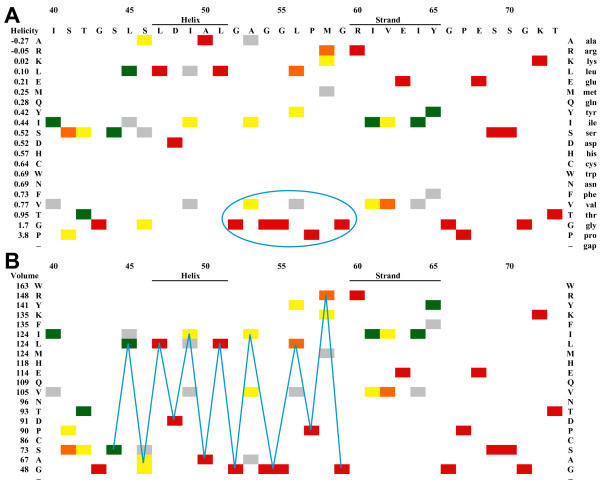
**Structural analysis of MAW and P-loop motif regions**. The MAW and P-loop motifs are highly conserved parts of the RecA protein family found at *E. coli *homolog positions 40–65 and 66–73, respectively. Labels denote the locations of α-helix B and β-strand 1 from the *E. coli *RecA crystal structure. Sorting the ProfileGrid rows by various amino acid physical constants reveals structural patterns within the context of the entire MSA. (A) Sorting by decreasing helical propensity shows that residues which do not favor helical formation (circled) immediately follow a helix in the MAW motif. (B) Sorting by decreasing volume displays the pattern (blue lines) that large amino acids are flanked by residues smaller than threonine. Whereas these panels were generated from the spreadsheet output, the JProfileGrid software allows an interactive analysis by switching between residue properties and color schemes.

The ProfileGrid in Figure 9 displays the MAW and P-loop motifs sorted by the residue properties of helicity and volume. Among RecA homologs, the region separating helix B and strand 1 is dominated by residues which do not favor helix formation (Figure 9A). The conserved glycines are probably necessary for the tight turn that occurs in this area [70]. Sorting the MAW motif ProfileGrid by amino acid sidechain volume (Figure 9B) allows the visualization of two other structural features. First, the loop from residues 41 to 44 is composed of small amino acids, namely threonine or smaller. Intriguingly, an *E. coli *RecA mutant with a change of 44-serine to the much larger leucine residue is proficient for *in vivo *recombination activity. However, the mutant is resistant to the recombination inhibitory effect of overexpression of the UmuD'C complex [71]. The second observed volume feature is that large residues between positions 45 and 58 are, in general, flanked on either side by small amino acids resulting in an alternating pattern of small-large-small residues.

When considering distant RecA homologs from all domains of life, the MAW motif is better conserved than the recently defined DNA interacting residues (Table 2). It is curious, then, that no clear function has been attributed to the MAW motif so here we speculate on possible roles. Universally conserved residues can be involved in ligand interactions or in protein folding [72-74]. While a ligand interacting role is a formal possibility for the MAW motif, this region of the protein forms part of the RecA hydrophobic core. However, one or more residues in the segment spanning positions 61–72 can be crosslinked to bound single-stranded DNA [75]. This suggests that parts of the MAW motif may not remain buried in the protein core at all times and that the motif may be involved in DNA binding. With respect to a protein folding role, the RecA ProfileGrid shows a high prevalence of isoleucine, leucine, and valine residues among bacterial RecA MAW motifs (Additional file 2). Specifically, two conserved leucines are on the same face of helix B (positions 47 and 51). Two properties of leucine may be relevant to this observation. First, in a study of crystal structures, leucine was found to have the largest amount of sidechain flexibility when buried [52]. Second, leucine is known to stabilize helices [76] which agrees with a theoretical study of RecA family helices. The residues from 44 to 51 of helix B have a near optimal sequence for thermostability when compared to other central domain helices [77]. Also, mutation of position 51 from leucine to phenylalanine results in a RecA mutant that is inactive for activities both *in vivo *and *in vitro *[78,79]. Thus, a role for the MAW motif may be to initiate protein folding or to stabilize the RecA protein core mediated by the motif structural features described above. Perhaps such a protein folding role is significant for a motif that connects an ATP binding site to the hinge region that undergoes conformational changes upon cofactor binding.

### Highlighting unique *B. subtilis *RecA residues

**Figure 10:**
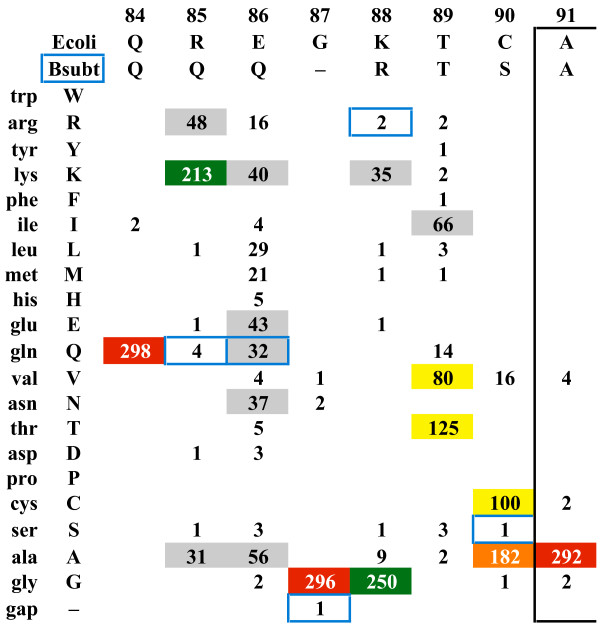
**Representing a unique *B. subtilis *RecA sequence feature**. In this ProfileGrid where the residues are sorted by volume, the *B. subtilis *RecA homolog is chosen as the "highlight sequence" and appears in the row immediately under the *E. coli *RecA template sequence. JProfileGrid performs a pair-wise comparison and represents any differences between the two sequences with blue boxes. It is clear within the context of the entire MSA that *B. subtilis *has a rarely occurring sequence from residues 85 to 90 (*E. coli *RecA numbering).

The JProfileGrid "highlight sequence" feature can draw attention to any unique residues of a particular sequence within the context of the entire MSA. Here, we analyze the *B. subtilis *RecA protein [GenBank:CAB13567.1]. The ProfileGrid of Figure 10 clearly shows that the characters 85-gln, 87-gap, 88-arg, and 90-ser are rarely found between the highly conserved positions 84 and 91. In addition, 88-arg is significantly larger than the more frequently observed glycine. Given the aforementioned caution about overinterpreting rare residues, we do not believe that the unique *B. subtilis *RecA feature described here is a due to a sequence error. We found the same result in two redundant *B. subtilis *RecA sequences determined from different research groups [GenBank:CAA36377.1, GenBank:AAB47709.1]. What could be the functional role for these residues? We note that there is controversy regarding the ability of the *B. subtilis *RecA protein to hydrolyze the cofactor ATP [80-82]. We suggest that this region of the *B. subtilis *RecA protein be targeted for site-directed mutagenesis to ascertain if this rare sequence feature influences a potentially unique biochemical activity.

## Conclusion

ProfileGrids serve as a new visual representation of large sequence alignments where the entire information content is presented in a concise form. The JProfileGrid Java software facilitates the creation and analysis of this alignment depiction. With the advent of sequence databases and software programs adopting MSA viewers, the traditional stacked sequence presentation is burdensome for large alignments especially for the interactive analysis of structural patterns and rare features. Thus, we anticipate that the ProfileGrid paradigm will have widespread application in bioinformatics. Finally, we describe and analyze a curated RecA protein data set whose representation as a ProfileGrid will serve as a valuable resource for researchers studying this ubiquitous protein.

## Availability and requirements

**Project name**: JProfileGrid version 1.1.1

**Project home page**: 

**Operating systems**: Platform independent

**Programming language**: Java 1.5 or higher

**License**: University of California license; see 

**Any restrictions to use by non-academics**: license required for commercial use

## Abbreviations

MSA: Multiple Sequence Alignment

## Competing interests

The authors declare that they have no competing interests.

## Authors' contributions

AIR designed the software, collected RecA sequences, performed the bioinformatic analysis & biocuration, and wrote the majority of the manuscript and documentation. AEA collected sequences. ACA wrote Java code and contributed to writing the manuscript and documentation. All authors read and approved the final manuscript and the response to reviewer comments.

## Supplementary Material

Additional file 1 **Multiple sequence alignment of bacterial RecA homologs**. A subset of the 300 sequences is shown representing each of the major bacterial phyla. In the alignment, a dash (-) indicates a gap and a period indicates an amino acid identical to the *E. coli *RecA protein. NCBI Protein database accession numbers are listed at the end unless the data was taken from the TIGR unfinished microbial genomes database. Summary lines above the alignment were calculated from all 300 sequences. The "Bioin" line indicates the bioinformatic structural elements (nanoanatomy) across the entire RecA protein: 12 motifs and the 10 connecting variable regions. "Secon" are the secondary structural elements from the *E. coli *RecA crystal structure where "a" are α helices, "b" are β strands, "l" are disordered loops, and "?" are disordered termini [62]. In each case the letter or number name of the element is given in the second position. "Ident" are the 21 resides identical in all 300 sequences. "Chemi" are the 39 chemically conservative substitutions based on the following amino acid classification: a = (DE), b = (HKR), f = (AGILV), m = (NQ), o = (FWY), h = (ST), i = (P), s = (CM). "Funct" lists the 55 functionally conservative residue substitutions based on the classification: a = (DE), b = (HKR), f = (AFILMPVW), p = (CGNQSTY). Finally, "Major" are the 187 residues conserved above a 70% majority threshold (210 sequences) with invariant residues shown in uppercase. The numbering of the alignment is based upon the *E. coli *RecA protein sequence. Click here for file

Additional file 2 **Detailed ProfileGrid of the RecA protein family**. The frequency values were calculated from the 300 RecA sequences over the full length (352 residues) of the *E. coli *RecA homolog (top sequence) that determines the position numbering. The "Major" summary line is the 187 residues conserved above a 70% majority threshold. The 12 RecA family motifs are boxed and labeled (as in Additional file 1) while the connecting variable regions are only labeled. Frequency values are shaded in the ranges of 50 to 69% (light gray), 70 to 89% (dark gray), and 90 to 100% (black). Since we anticipate updating the analysis in the future, this is version 1.0 of the RecA ProfileGrid. Click here for file
