# Supplementary material for: ProfileGrids as a new visual representation of large multiple sequence alignments: a case study of the RecA protein family
Source: BMC Bioinformatics. 2008 Dec 22;9:554. doi: 10.1186/1471-2105-9-554 (PMC2663765; doi:10.1186/1471-2105-9-554)
Supplement: Additional file 1 — Multiple sequence alignment of bacterial RecA homologs. A subset of the 300 sequences is shown representing each of the major bacterial phyla. In the alignment, a dash (-) indicates a gap and a period indicates an amino acid identical to the E. coli RecA protein. NCBI Protein database accession numbers are listed at the end unless the data was taken from the TIGR unfinished microbial genomes database. Summary lines above the alignment were calculated from all 300 sequences. The "Bioin" line indicates the bioinformatic structural elements (nanoanatomy) across the entire RecA protein: 12 motifs and the 10 connecting variable regions. "Secon" are the secondary structural elements from the E. coli RecA crystal structure where "a" are α helices, "b" are β strands, "l" are disordered loops, and "?" are disordered termini [62]. In each case the letter or number name of the element is given in the second position. "Ident" are the 21 resides identical in all 300 sequences. "Chemi" are the 39 chemically conservative substitutions based on the following amino acid classification: a = (DE), b = (HKR), f = (AGILV), m = (NQ), o = (FWY), h = (ST), i = (P), s = (CM). "Funct" lists the 55 functionally conservative residue substitutions based on the classification: a = (DE), b = (HKR), f = (AFILMPVW), p = (CGNQSTY). Finally, "Major" are the 187 residues conserved above a 70% majority threshold (210 sequences) with invariant residues shown in uppercase. The numbering of the alignment is based upon the E. coli RecA protein sequence. [file 1471-2105-9-554-S1.pdf]

```
Bioin     Variable 0    |--Motif-0---|   Var 1   |---------Mot-1a----------|--Mot-1b--|  Var 2  |----Mot-2------
Secon   ??aAaaaaaaaaaaaaaaaaa____b0b________--________aBaaaaa_________b1bbbb_____aCaaaaaaaaaaaaa__b2bbbb______aD
Ident   ....................................--..................G...G...E..G.E..GK.T...................D........
Chemi   ....................................--.....h............f...fb..af.f.a..fbhh..................fa........
Funct   ....................................--.....p....f.......p.f.pbf.af.p.a..pbppf.................fa........
Major   ........al..a...iek.fgkg..m.lg...........i.tgsl.ld.alg.gG.p.Gri.EiyGpEssGKtT..l...a..qk.gg..afiDaehaldp.
                 10        20        30          40        50        60        70        80        90       100
        ---------+---------+---------+------  ---+---------+---------+---------+---------+---------+---------+--

Ecoli   AIDENKQKALAAALGQIEKQFGKGSIMRLGEDRSMD--VETISTGSLSLDIALGAGGLPMGRIVEIYGPESSGKTTLTLQVIAAAQREGKTCAFIDAEHALDPI
Mtube   TQTPDRE...EL.VA....SY....V....DE--ARQPISV.P...IA..V...I....R..VI............VA.HAV.N..AA.GVA...........D
Apyro   ENLSE.M...EV..SS...R....AV.P.KAV--ETVE....P...I.....T.V..I.K...T..F.V........A.H...E..KR.GVAV..........K
Bfrag   -------------MDK...S.......KM..E--VVEQ..V.P...IA.NA...V..Y.R...I.............AIHA..E..KA.GIA........F.RF
Ctrac   MSVPDRKR..E..IAY......A....S..KHS-SAHEIS..K..A....L...I..V.K......F..........ATHIV.N..KM.GVA.Y.........N
Dethe   -MTTE.D...ELTV.I...R.......K.SDPT-FRQT..F.P.S..A......V..I.R...S..F...G......AQHI..Q..KM.EKA.Y..V......K
Avari   TDTSG.....TMV.N...RS....A.....DA--TRMR.......A.T..L...-....R..VI............VA.HA..EV.K..GIA..V...Q....T
Dradi   TDAKERS..IET.MS....A.......K..AES--KLD.QVV........L...V..I.R...T......G......A.AIV.Q..KA.G.............V
Dther   ----E.E...ES.IF.........A.....S--QERFQ.PV.P..I.T..Y...V..V.R...I.LF...G.....VA..I..S..KM.GVA........F..N
Fsucc   ----------------...NY......A..QQ--PVEDIPV.P..CIQ..M...V..F.R...I.............A.HA..E..KL.GVA........F.AV
Bsubt   --MSDR.A..DM..K............K...K--T.TRIS.VPS...A..T...I..Y.R...I.V..........VA.HA..EV.QQ-R.S...........V
Fnucl   TDK.G.E..VKD.MAA.T.G..S.L..K...K--SSMN..S.P...IN......I..V.K...I....A........A.H...E..KQ.G.V...........V
Tyell   ---KD.L...EI.IS....N....A.....TK-AHAEGIGV.P...I.....T.I..Y.R..VI..F..........A.HA..E...H.GVA..........VN
Gobsc   GGSIKDN.E.KT..AA...E.......S..DMN--GLDIDC....A....L...GK.I.R........A.A.....IA.HT..Q..KA.GVA...........S
Rcaps   KGKAD.....EC..A...R........K..G.TPPPE-I.AT.....G......I....K...................HC..EE.KK.GV...V........Y
Ngono   -MSDD.S.......A....S....A..KMDGS-QQEENL.V......G..L...V....R......F..........C.EAV.QC.KN.GV...V.....F..V
Cjeju   -M.D..R.S.D...KSLD.T....T.L...DK--EVEQIDS.G...VG..L...I..V.K...I...............HI..EC.KA.GV...........VK
Bburg   IERAS.EE.IEL.RV....A.....LIKM..SP-VGQGIKSM.S..IV..E...I..Y.R...I..F.............A..EV.K..GIA...........V
Tcomm   ----E.K..VE..IS....M...........G-AKKIE.SV.P.........T.I..I.R...T....A.A......A.HMV.E..KQ.GVA..........LN
Tmari   PEEKQ.KSV.EK..KR..EN.......I..DETQVQP-..V.P...AI....T.V..Y.R......F.Q........A.HA..E..KM.GVA...........V


Bioin   -|  Var 3  |----Mot-3----|  Var 4   |--------Mot-4a--------|----Mot-4b------|   Var 5   |----Mot-5a----|
Secon   aDaaa______b3bbb___aEaaaaaaaaaaaa____b4bbb____________l-1llllllaFaaaaaaaaaaaaaaaaaaa_b5bbbbbl2lllllll-ll
Ident   ...................GE....................DSV.A.........-..........A........................NQ........-..
Chemi   o.....f............fa..................ffahf.f.....a...-..........fb.......................mm........-..
Funct   ......f....f.......pa..f.f...f.....f.ffffapf.ff....a...-..........fbff..f...............f..pp....fp..-..
Major   ya..lgv....ll.sqpd.GEqalei...l.rsga.d..v.DSVaAl.p.aeieg-emgd...glqArlmsqalrkl.g......t..ifiNQ.r.kigvm-fg
               110       120       130       140       150        160       170       180       190       200
        -------+---------+---------+---------+---------+-------  --+---------+---------+---------+---------+-- --

Ecoli   YARKLGVDIDNLLCSQPDTGEQALEICDALARSGAVDVIVVDSVAALTPKAEIEG-EIGDSHMGLAARMMSQAMRKLAGNLKQSNTLLIFINQIRMKIGVM-FG
Mtube   ..K.....T.S..V............A.M.I....L.IV.I......V.R..L..-.M....V..Q..L....L..MT.A.NN.G.TA.....L.D.....-..
Apyro   ..K.....V...YI....Y.......AES.IN...............V..D.L..-.M.EAQV.KQ..L....L...K.AVHR...A........E.....-..
Bfrag   ..A.....V...FI....N.......AEQ.I..S.I.I.................-DM..NKV..Q..L....L...TSAVSKTR.TC.....L.E.....-..
Ctrac   ..ALI.AN.ND.MI....C..D..S.AEL...........I......V..S.L..-....V.V..Q.......L...TAT.ART..CA.......E....S-..
Dethe   ..STC..NL.E..I.......E..G.AEE.V..T.IG...I......V.......-DM....V..Q..L....L...TASIG.TR.AV.....L.E.V...-..
Avari   ..SA.....Q...V.......S....V.Q.VP.A...IV.I......V.R.....-DM..A.V..Q..L....L..IT..IGK.GCTV.....L.Q....T-Y.
Dradi   ...A...NT.E..V....N.......MEL.V....I..V..........R.....-DM...LP..Q..L....L...TAI.SKTG.AA.....V.E.....-Y.
Dther   ....I..NLE...I............AEI.V...G.....I......V.R..L..-.M..AFI..Q..L....L...T.VISK.K.VA.....L.E.V.-VF..
Fsucc   .........ES..V..........D.AET.V....I.I.........V.Q...N.-.M..N.V..Q..L....L...T.I.SK...CML....L.......-..
Bsubt   ..Q....N.EE..L............AE..V......IV........V.......-DM....V..Q..L....L...S.AINK.K.IA.......E.V...-..
Fnucl   ..KA......E..I....Y.......A.T.V....I.L..I......V.....D.-.MS.QQ...Q..L..KGL...T...NKYK.TM.......E....T-Y.
Tyell   ..S.....VE...I...........VTET.V......I..I......V.......-.M...LP..Q..L....L...TAAISK.Q.AV.......Q...-VM..
Gobsc   W.KR....L....V...GY..E..R.AEM.VK.N...L.........V..N..QDS....TKV..Q..L......I.TPQINK.R.C.M......Q.....-Y.
Rcaps   ..K....SLED..I............V.T.V....SLV.................-DM..ATV.AQ..L........TASIGR..CMV.............-..
Ngono   .......KVEE.YL..............T.V...GI.MV........V.......-DM....V..Q..L....L...T.HI.KT...VV............-..
Cjeju   ..KN...NT.D.YV....F.......VETI.......L.................-DM..Q.V..Q..L....L...T.IVHKM..TV...........A.GY.
Bburg   ..KA...NVAE.WL............AEH.I...G..L............L..D.-.M...QI..Q..L..K.L..IT.I.SK...CIM......R.....-..
Tcomm   ..Q....N.ED..V............AEV........I..I......V.......-.ME.QQV..Q..L..K.....TSAISK...AVV....T.....TFSY.
Tmari   ..KN....LKS..I....H.......V.E.V...V..L.........V.R.....-AM..MQV..Q..L....L..I..SVNK.KAVV..T..........-..
```

```
Bioin   |-------Mot-5b---------|     Var 6        |------Mot-6a------|--Mot-6b-|     Var 7      |----Mot-7-----|
Secon   -lllll___aGaaaa__b6bbbbb______-___---____b7bbbb___-______b8bbbbb___-____aHaaaaaaaa___b9bb__b10b_________
Ident   -......G.....F................-...---...........K.-................-....................................
Chemi   -......ff.ffboo.....a.........-...---...........b.-................-.f..................................
Funct   -......p...fbf......a.........-...---...........bp-................-.........f........f.................
Major   -.petttGg.alkFyasvrld.rr....k.-g..---..g..t..kvvKn-kvappf..ae.di.yg-.gis..ge..d..v......k.g.w.sy.....gqg
              210       220       230           240        250       260        270       280       290       300
         -----+---------+---------+--- ---   ---+--------- +---------+----- ----+---------+---------+---------+-
Ecoli   -NPETTTGGNALKFYASVRLDIRRIGAVKE-GEN---VVGSETRVKVVKN-KIAAPFKQAEFQILYG-EGINFYGELVDLGVKEKLIEKAGAWYSYKGEKIGQG
Mtube   -S.......K.........M.V..VETL.D-.T.---A..NR........-.CSP.......D....-K..SRE.S.I.M..DQG..R.S...FT.E..QL...
Apyro   -.....P..R....FSDM..EV..L.D...G..K----K.YRVK.R....-.L.P..QE...DVI..-...CRICDII.TAANLGV.T.S.S....GEKRL...
Bfrag   -......................GSQQI.D-..E---.I.KQ.K......-.V.P..RK...D.MF.-...SHS..II...ADLGI.K.S.S....NDT.L...
Ctrac   -........R.....S.I.I......SI.GGENF---DI.NRIK...A..-.L.P..RT...D..FN-...SSA.CII..A.EKNI.D.K.S.FN.QDR.L...
Dethe   -...V.P..R.....S...I.L...ETI.Q-.TV---AI.TRV.A.....-.V.P..RT...D.MFD-S..SRE.N.I....TSEV.R....FF..GDIRL...
Avari   -S.......................QTL.K-.TD---EF.NRVK...A..-.V.P..RI...D.IF.-K.VSTL.C....AEETGILLRK......N.DN.S..
Dradi   -........R...........V.K..QPTKV.ND---A.ANTVKI.T...-.V.....EV.LALV..-K.FDQLSD..G.AADMDI.K...SF...GD.R....
Dther   -.....P..R.........I.V.KAESM..-.SE---.T.TKVK......-.V.P...EG..ELI..-...SRE.CIL.A..EA.V..RS.T..I..DIRL...
Fsucc   -...............TQ.I.....A.I.N-..E---.I.NR....I...-.V....T.C..D....-V..SREASIL..ACELDI.Q.S.S.F..NN.R....
Bsubt   -.....P..R.....S....EV..AEQL.Q-.ND---.M.NK.KI.....-.V.P..RT..VD.M..-...SKE..II...TELDIVQ.S.S....EE.RL...
Fnucl   -PTT.....K.....S...MEVKKM.T..Q-.DD---PI...VI...T..-.V.P...E.A.E....-K..SKV..II.AA.AKDV.V...S.F.FRDQS....
Tyell   -.....P..T.......M.....K.DTL..-.QE---TT.GRV...I...-.V.P.......D.YFN-...SKT..IL..A.EKGI...S....N.N.SRLA..
Gobsc   -D.N.....L.........MEVK.VTH..D-..D---TI.A....R....-...P..RN...EL.HD-R..D.E.DVIK.ALEDEV...S..HF...EQRL...
Rcaps   -S....S.................T..I.D-RDE---.I.NQ........-.V.P..REV..D....-...SKV.........AGVVA.S......GD.R....
Ngono   -S.............S........T.SI.K-..E---.L.N......I..-.V.P..R....D....-...SWE...I.I...NDI.N.S......N.A.....
Cjeju   -T...................V.KVATL.Q-N.E---PI.NRVK......-.V.P..R....DVMF.-..LSRE...I.Y...LDIVD.S...F...DK.L...
Bburg   -..............S.L..EV.K.EQ.TRS.S-SDD.I.NKI...I...-.V.P..RKV.LI.YF.-K..SREAGIL.AAI.HN..Q.T.S...LGDN.L...
Tcomm   GP....P..M....F..L..E.KK.ASI..-.QE---PL.HRVK......-.L.P...EV..D.YF.-...SKEA.......EMEV...S.S.F..Q..RL...
Tmari   -S.......L......TM.MEV..GEPI..-.KD---.I.NVIS..I...-.V.P...T.QTY.I..-K..DREY..FNIA.N.GIVDRK.S..Y.TTL.GEEV


Bioin             Var 8a      |-----------Var-8b----------
Secon   aIaaaaaaaa__aJaaaaaaaaaaaaaa????????????????????????
Ident   ...................................................
Chemi   ...................................................
Funct   ...................................................
Major   ..n....l........e..................................
                310       320       330       340       350
        --------+---------+---------+---------+---------+--
Ecoli   KANATAWLKDNPETAKEIEKKVRELLLSNPNSTPDFSVDDSEGVAETNEDF     Escherichia coli                    AAC75741.1
Mtube   .E..RNF.VE.ADV.D.....IK.K.GIGAVV.D.P.N.GVLPAPVDF        Mycobacterium tuberculosis          CAA15533.1
Apyro   REQ.KKY.LEH..MLE...R....VSGLVRPD.ENSVGEK..              Aquifex pyrophilus                  AAA67702.1
Bfrag   RDA.KQCIA....L.E.L.GLIF.K.REHK                          Bacteroides fragilis                AAA22918.1
Ctrac   REAVREE..R.K.LFH.L.RRIY.SVQASQAPAAACVDSE.RE...AAK       Chlamydia trachomatis               AAA75588.1
Dethe   RES.KNY.AA..DL.Q...E.I.ASAVTLCSIGDGD                    Dehalococcoides ethenogenes         AAW39122.1
Avari   RD..IKY.EEK..F.EQ.KQQ...K.DKGAVVSANSVAKAN.EDE.DVDLD     Anabaena variabilis                 AAA22031.1
Dradi   .EKTI.YIAER..MEQ..RDR.MAAIRAGNAGEAPALAPAPAAPEAAEA       Deinococcus radiodurans             AAF11887.1
Dther   RE..KLY..EH..I.D...REI.KIWGFEEITVENNN.KEEPKEEK.GKKA     Dictyoglomus thermophilum           TIGR UFMG
Fsucc   RE.TRLF....A.LCS...Q.I..SMKD                            Fibrobacter succinogenes            TIGR UFMG
Bsubt   RE..KQF..E.KDIMLM.QEQI..HYGLDN.GVVQQQAEETQEEL.FE.       Bacillus subtilis                   CAB13567.1
Fnucl   .EKVR.E.EI...LLAQV..DLK.AIAKG.VDKKKKKSKKEASSDD.DDEN     Fusobacterium nucleatum             AAL94743.1
Tyell   RE..KEY..TH..IFN..YS..LDVYGLKQPKVSEGENKTE               Thermodesulfovibrio yellowstonii    TIGR UFMG
Gobsc   LK..VEF.RE.HAVRD..VAA.K.KRTPKVVDAAALEATAAQEE..AAA       Gemmata obscuriglobus               TIGR UFMG
Rcaps   RE..KQF.R...DI.Y...D.I.ASHGLEFGVD.TAEDLTE.              Rhodobacter capsulatus              CAA57673.1
Ngono   .D.VRV...E...ISD..DA.I.A.NGVEMHI.EGTQDETDGERP.E         Neisseria gonorrhoeae               CAA35247.1
Cjeju   RE.SK.F..E...I.D..T.AIQNSMGIEGMISGSEDDEGE.              Campylobacter jejuni                CAB73660.1
Bburg   RESVIEY.SKEV.L.NNLD.RL.KIIFN.FDQEN.NFIEFK.DES.          Borrelia burgdorferi                AAC66507.1
Tcomm   .E.VRKL..E.KQL.L...N.I..KAGLPLIK..SEKSTEKPQKGKQ         Thermodesulfobacterium commune      TIGR UFMG
Tmari   SLGQGSSNAVQFLKDNPEIAGEI.RRIREKYGLLSVEKEEQRKEKKSSGEE     Thermotoga maritima                 AAD36921.1
```

Additional File 1. Multiple sequence alignment of bacterial RecA homologs. A subset of the 300 sequences is shown representing each of the major bacterial phyla. In the alignment, a dash (-) indicates a gap and a period (.) indicates an amino acid identical to the E. coli RecA protein. NCBI Protein database accession numbers are listed at the end unless the data was taken from the TIGR unfinished microbial genomes (UFMG) database. Summary lines above the alignment were calculated from all 300 sequences. The “Bioin” line indicates the bioinformatic structural elements (nanoanatomy) across the entire RecA protein: 12 motifs (0 thru 7) and the 10 connecting variable regions (0 thru 8b) that connect the motifs. “Secon” are the secondary structural elements from the E. coli RecA crystal structure where “a” are α helices, “b” are β strands, “l” are disordered loops, and “?” are disordered termini (Story, Weber, & Steitz, 1992). In each case the letter or number name of the element is given in the second position. “Ident” are the 21 resides identical in all 300 sequences. “Chemi” are the 39 chemically conservative substitutions based on the following amino acid classification: a=(DE), b=(HKR), f=(AGILV), m=(NQ), o=(FWY), h=(ST), i=(P), s=(CM). “Funct” lists the 55 functionally conservative residue substitutions based on the classification: a=(DE), b=(HKR), f=(AFILMPVW), p=(CGNQSTY). Finally, “Major” are the 187 residues conserved above a 70% majority threshold (210 sequences) with invariant residues shown in uppercase. The numbering of the alignment is based upon the E. coli RecA protein sequence.

© Roca 2008
