# Supplementary material for: ProfileGrids as a new visual representation of large multiple sequence alignments: a case study of the RecA protein family
Source: BMC Bioinformatics. 2008 Dec 22;9:554. doi: 10.1186/1471-2105-9-554 (PMC2663765; doi:10.1186/1471-2105-9-554)
Supplement: Additional file 2 — Detailed ProfileGrid of the RecA protein family. The frequency values were calculated from the 300 RecA sequences over the full length (352 residues) of the E. coli RecA homolog (top sequence) that determines the position numbering. The "Major" summary line is the 187 residues conserved above a 70% majority threshold. The 12 RecA family motifs are boxed and labeled (as in Additional file 1) while the connecting variable regions are only labeled. Frequency values are shaded in the ranges of 50 to 69% (light gray), 70 to 89% (dark gray), and 90 to 100% (black). Since we anticipate updating the analysis in the future, this is version 1.0 of the RecA ProfileGrid. [file 1471-2105-9-554-S2.pdf]

| | | 1 | | | | 5 | | | | | 10 | | | | | 15 | | | | | 20 | | | | | 25 | | | | | 30 | | | | | 35 | | | | | | 39 | |
|---|---|---|---|---|---|---|---|---|---|---|---|---|---|---|---|---|---|---|---|---|---|---|---|---|---|---|---|---|---|---|---|---|---|---|---|---|---|---|---|---|---|---|---|
| | | | | | | | Variable 0 | | | | | | | | | | | | | | | | Motif 0 | | | | | | | | | | | | | | Variable 1 | | | | | | |
| Major | | . | . | . | . | . | . | . | . | a | l | . | . | a | . | . | . | i | e | k | . | f | g | k | g | . | . | m | . | l | g | . | . | . | . | . | . | . | . | . | . | . | |
| Ecoli | | A | I | D | E | N | K | Q | K | A | L | A | A | A | L | G | Q | I | E | K | Q | F | G | K | G | S | I | M | R | L | G | E | D | R | S | M | D | - | - | V | E | T | |
| ala | A | 52 | 14 | 47 | 21 | 8 | | 6 | 39 | 264 | | 77 | 155 | 267 | | 83 | 5 | | | | 19 | | 1 | 7 | 1 | 74 | 2 | | 1 | 1 | | 20 | 23 | 2 | 20 | 52 | 24 | 11 | 3 | 1 | 5 | 48 | A |
| cys | C | | | | | | | | | | | | 1 | | | 1 | | | | | | | | | 1 | | | | | | | | | | | | 1 | 1 | | | | 2 | C |
| asp | D | 7 | 15 | 86 | 55 | 119 | 1 | 7 | | | | 82 | 14 | 1 | | 3 | | | 25 | | 21 | | | 1 | | 1 | 1 | 2 | | | 6 | 131 | 34 | 14 | 5 | 3 | 67 | 9 | 30 | | 32 | | D |
| glu | E | 7 | 7 | 10 | 69 | 56 | 1 | 50 | 2 | 5 | | 81 | 2 | | | 5 | 6 | | 260 | 3 | 4 | | | 4 | 3 | | | | 1 | 1 | 4 | 94 | 38 | 18 | 14 | 14 | 36 | 68 | 16 | | 119 | | E |
| phe | F | | 8 | 1 | | 1 | | | | | | | 1 | | | 1 | | 2 | | | | 249 | | | | | 3 | 1 | 2 | 2 | | | | | | 1 | | 2 | | | | 1 | F |
| gly | G | 8 | 2 | 21 | 4 | 4 | | | 1 | 2 | | 2 | 2 | | | 49 | | | 1 | | 1 | | 293 | | 286 | | | | | | 274 | 7 | 15 | 5 | 1 | 3 | 8 | 1 | 4 | | 1 | | G |
| his | H | | | | | | | | | | | | | | | | | | 1 | | | 3 | | | | | | 1 | 1 | | | 1 | 25 | | 2 | 1 | 2 | 10 | 2 | | | | H |
| ile | I | 4 | 26 | 2 | 7 | 2 | 3 | 2 | 1 | 1 | 28 | | 2 | 1 | 30 | | 1 | 279 | 2 | | 1 | | | | | 1 | 183 | 3 | 3 | | | | 1 | | | 21 | 11 | 2 | | 169 | | 4 | I |
| lys | K | 21 | 8 | 8 | 9 | 6 | 166 | 67 | 209 | 2 | 1 | 9 | 4 | 1 | | 73 | 30 | | 2 | 230 | 8 | | | 273 | | | | | 83 | | 3 | 11 | 51 | 3 | 3 | 9 | 18 | 8 | 36 | | 2 | | K |
| leu | L | 1 | 8 | 6 | 3 | | | 12 | 4 | | 255 | 2 | 51 | | 207 | 3 | 7 | 10 | | | | | | 3 | | 2 | 14 | 9 | 4 | 226 | | | | | 1 | 7 | 3 | 12 | | 7 | | | L |
| met | M | 21 | 84 | 20 | 17 | 2 | 1 | 2 | | | | | 18 | | 37 | 1 | 2 | 2 | | | 3 | | | | | | | 272 | | 62 | | | 5 | | | 39 | 5 | 14 | | 4 | | | M |
| asn | N | 6 | 15 | 10 | 20 | 71 | 3 | 10 | 1 | 1 | 2 | 11 | 6 | | 2 | 4 | 12 | | | | 39 | 2 | 2 | 1 | 2 | 1 | | | 2 | 1 | 3 | 2 | 35 | 4 | 6 | 6 | 12 | 1 | 24 | 1 | 3 | | N |
| pro | P | 6 | 5 | 5 | 37 | | | | 2 | 1 | | | | 1 | | | | | | | | | | | | | | | 3 | | | | 2 | 9 | 6 | 9 | 3 | 9 | 36 | | 16 | | P |
| gln | Q | 1 | 21 | 6 | 5 | 9 | | 74 | 5 | 1 | | 4 | 12 | | | 3 | 208 | | 3 | 2 | 140 | | | 2 | | | | | 2 | | | 13 | 13 | 4 | 13 | 25 | 12 | 71 | 25 | | 42 | | Q |
| arg | R | 3 | 4 | 2 | 1 | | 113 | 17 | 21 | | | 1 | | | 4 | 2 | 2 | | | 60 | 4 | | | 2 | | | | | 183 | | | 2 | 30 | 29 | 17 | | 43 | 7 | 19 | | | | R |
| ser | S | 12 | 15 | 24 | 20 | 5 | | 34 | 2 | 9 | | 18 | 6 | | | 61 | 11 | | | | 43 | | | 2 | 1 | 206 | | 1 | 8 | 1 | 8 | 16 | 14 | 15 | 23 | 26 | 5 | 2 | 2 | | 78 | 19 | S |
| thr | T | 13 | 6 | 18 | 9 | 1 | 1 | 7 | 1 | 1 | | 4 | 14 | 7 | 2 | 4 | 5 | | 2 | 1 | 8 | | | 1 | 2 | 11 | | | 3 | | | 1 | 5 | 9 | 6 | 15 | 6 | 10 | 8 | 2 | | 112 | T |
| val | V | 5 | 7 | 6 | 6 | | | | 1 | 2 | 5 | | 4 | 14 | 13 | 2 | | 3 | | | 5 | | | | | | 92 | 8 | 2 | 1 | | | 4 | | 10 | 55 | 41 | 4 | | 114 | | 112 | V |
| trp | W | | | | | 2 | | 1 | | | | | | | | | | | | | | | | | | | | | | | | | | | | | | | | | | | W |
| tyr | Y | | 2 | | | | | | | | | 1 | | | | | 6 | | | | | 42 | | | | | 1 | 1 | | 3 | | | | | | | | | 3 | | | | Y |
| gap | – | 133 | 53 | 28 | 17 | 14 | 11 | 11 | 11 | 11 | 9 | 8 | 8 | 8 | 5 | 5 | 5 | 4 | 4 | 4 | 4 | 4 | 4 | 4 | 4 | 4 | 4 | 2 | 2 | 2 | 2 | 2 | 5 | 188 | 173 | 14 | 3 | 58 | 92 | 2 | 2 | 2 | – |

| | | 40 | | | | | 45 | | | | | 50 | | | | | 55 | | | | | 60 | | | | | 65 | | | | | 70 | | | | | 75 | | | | | 80 | | | | 84 | |
|---|---|---|---|---|---|---|---|---|---|---|---|---|---|---|---|---|---|---|---|---|---|---|---|---|---|---|---|---|---|---|---|---|---|---|---|---|---|---|---|---|---|---|---|---|---|---|---|
| | | | | | | | | | | | | | | Motif 1a | | | | | | | | | | | | | | | | | | Motif 1b | | | | | | | | | | Variable 2 | | | | | |
| Major | | i | . | t | g | s | l | . | l | d | . | a | l | g | . | g | G | . | p | . | G | r | i | . | E | i | y | G | p | E | s | s | G | K | t | T | . | . | l | . | . | . | a | . | . | q | |
| Ecoli | | I | S | T | G | S | L | S | L | D | I | A | L | G | A | G | G | L | P | M | G | R | I | V | E | I | Y | G | P | E | S | S | G | K | T | T | L | T | L | Q | V | I | A | A | A | Q | |
| ala | A | | | | 1 | 24 | | 108 | | | 6 | 298 | | | 40 | | | | | | | | | | | | | | 5 | | 3 | 1 | | | | | | 163 | 1 | 1 | 152 | 2 | 295 | 29 | 169 | | A |
| cys | C | | | | | 4 | | | | | 1 | | | | 1 | | | | | 1 | | | | 1 | | | | | | | | 1 | | | | | | 6 | | | 5 | | 2 | | 30 | | C |
| asp | D | | | | | | | 5 | | 296 | 5 | | | | | 1 | | | | | | | | | | | | | | | | | | | | | | | | | | | | | | | D |
| glu | E | | | | | | | 1 | | | 6 | | | | | | | | | | | | | | 300 | | | | | 300 | | | | | | | | | | 14 | | | | 177 | 3 | | E |
| phe | F | 5 | | | | | | | 1 | | | | | | | | | 13 | | | | | | | | | 43 | | | | | | | | | | | | | | | | | | 2 | | F |
| gly | G | | 1 | | 297 | | | 89 | | | | | | 298 | 2 | 292 | 300 | | | | 300 | | | | | | | 300 | | | 2 | 5 | 300 | | | | | | | | | 1 | | | | | G |
| his | H | | | | | | | 2 | | | 2 | | | | | 2 | | | | | | | | | | | | | | | | | | | | | | | | 192 | | | | | | | H |
| ile | I | 219 | | | | 3 | 53 | | 10 | | 148 | 1 | 3 | 1 | 131 | | | 17 | 1 | 2 | | | 218 | 128 | | 240 | | | | | | | | | | | 8 | | 5 | | 37 | 204 | | | 6 | 2 | I |
| lys | K | | 8 | | | | | 1 | | | | | | | | 2 | | | | 106 | | 2 | | | | | | | | | | | | 300 | | | | | | | | | 2 | | | | K |
| leu | L | 1 | | | | | 226 | 7 | 286 | | 46 | | 287 | | | | | 151 | | 2 | | | 1 | | | 4 | 1 | | | | | | | | | | 196 | | 285 | | 11 | 2 | | | 6 | | L |
| met | M | 13 | | | | | 1 | 6 | 1 | | 5 | | 1 | | | | | 1 | | 31 | | | 1 | 1 | | | | | | | | | | | | | 3 | | 1 | | 2 | 1 | | | 15 | | M |
| asn | N | | | | | 6 | | 3 | | 4 | 5 | | | | | 3 | | | | | | | | | | | | | 6 | | | | | | | | | | | 1 | | | | 38 | | | N |
| pro | P | | 108 | | | | | | | | | | | | | | | | 299 | | | | | | | | | | 286 | | | | | | | | | | | | | | | | | | P |
| gln | Q | | | | | | | 1 | | | 3 | | | | | | | | | 1 | | | | | | | | | 1 | | | | | | | | | | 2 | 63 | | | | 45 | | 298 | Q |
| arg | R | | 1 | | 1 | | | | | | 1 | | | | | | | | | 154 | | 298 | | | | | | | | | | | | | | | | | | | | | | | | | R |
| ser | S | 1 | 182 | 86 | 1 | 263 | | 58 | | | 3 | | | 1 | 3 | | | | | 2 | | | | 1 | | | | | | | 295 | 293 | | | 1 | | | 15 | | 25 | 1 | | | 11 | 4 | | S |
| thr | T | 5 | | 214 | | | 10 | 17 | | | 2 | 1 | 8 | | 4 | | | | | | | | | 11 | | | | | | | | | | | 299 | 300 | | 116 | 6 | 4 | 16 | | | | 5 | | T |
| val | V | 55 | | | | | 9 | 1 | 2 | | 63 | | | | 107 | | | 38 | | 1 | | | 80 | 158 | | 56 | | | 2 | | | | | | | | 93 | | | | 76 | 90 | | | 60 | | V |
| trp | W | | | | | | | | | | | | 1 | | | | | | | | | | | | | | | | | | | | | | | | | | | | | | | | | | W |
| tyr | Y | 1 | | | | | 1 | 1 | | | 4 | | | | | | | 80 | | | | | | | | | 256 | | | | | | | | | | | | | | | | 1 | | | | Y |
| gap | – | | | | | | | | | | | | | | 12 | | | | | | | | | | | | | | | | | | | | | | | | | | | | | | | | – |

| % | | sequences |
|---|---|---|
| 0 | | 0 |
| 10 | | 30 |
| 25 | | 75 |
| 50 | 69 | 150 209 |
| 70 | 89 | 210 269 |
| 90 | 100 | 270 300 |

© Roca 2008

| | | 85 | | | | | 90 | | | | | 95 | | | | | 100 | | | | | 105 | | | | | 110 | | | | | 115 | | | | | 120 | | | | | 125 | | | | 129 | |
|---|---|---|---|---|---|---|---|---|---|---|---|---|---|---|---|---|---|---|---|---|---|---|---|---|---|---|---|---|---|---|---|---|---|---|---|---|---|---|---|---|---|---|---|---|---|---|---|
| | | | | | | | | | | | | Motif 2 | | | | | | | | | | | | | Variable 3 | | | | | | | | | | | | | Motif 3 | | | | | | | | | |
| Major | | k | . | g | g | . | . | a | f | i | D | a | e | h | a | l | d | p | . | y | a | . | . | l | g | v | . | . | . | . | l | l | . | s | q | p | d | . | G | E | q | a | l | e | i | . | |
| Ecoli | | R | E | G | K | T | C | A | F | I | D | A | E | H | A | L | D | P | I | Y | A | R | K | L | G | V | D | I | D | N | L | L | C | S | Q | P | D | T | G | E | Q | A | L | E | I | C | |
| ala | A | 31 | 56 | | 9 | 2 | 182 | 292 | | | | 294 | | | 292 | | | 2 | 31 | | 294 | 42 | 34 | | | 5 | | 1 | 1 | 11 | | | | 12 | | | | 5 | | | 1 | 254 | | 2 | | 148 | A |
| cys | C | | | | | | 100 | 2 | | | | | | 1 | | | | | | | 2 | | | 2 | | | | 1 | | | | | 23 | 1 | | | | 6 | | | | | | | | 48 | C |
| asp | D | 1 | 3 | | | | | | | | 300 | | | | | | 297 | | 17 | | | 1 | 2 | | | | 136 | | 204 | 65 | | | | | | | 289 | | | | 6 | 1 | | 7 | | | D |
| glu | E | 1 | 43 | | 1 | | | | | | | | 299 | | | | 2 | | 32 | | | 15 | | | | | 1 | | 44 | 96 | | | | | 1 | | 2 | | | 300 | 13 | | | 271 | | | E |
| phe | F | | | | | 1 | | | 273 | | | | | 1 | 1 | 17 | | 1 | 4 | | | | | | | | | | | | | 4 | 1 | | | | | 9 | | | | | 6 | | | | F |
| gly | G | | 2 | 296 | 250 | | 1 | 2 | | | | 1 | | 3 | | | | | 6 | | | 15 | | | 298 | | | | 3 | 1 | | | | | | | 1 | | 300 | | | 45 | | 1 | | | G |
| his | H | | 5 | | | | | | | | | | | 284 | | | | | 3 | | | 2 | 8 | | | | | | | | | | | | 3 | | | 9 | | | | | | 1 | | | H |
| ile | I | | 4 | | | 66 | | | 2 | 234 | | | | | | 3 | | 6 | 36 | | | | | 14 | | 9 | | 135 | | | | 22 | 91 | | | | | | | | | | 1 | | 295 | 5 | I |
| lys | K | 213 | 40 | | 35 | 2 | | | | | | | 1 | | | | | 1 | 11 | | | 114 | 194 | | 1 | | 10 | | 3 | 8 | | | | | | | 1 | | | | | | | | | | K |
| leu | L | 1 | 29 | | 1 | 3 | | | 2 | 1 | | 1 | | | | 274 | | 4 | 5 | | 1 | | 6 | 283 | | | | 34 | | | 299 | 241 | 77 | | | | | | | | | | 291 | | 2 | 9 | L |
| met | M | | 21 | | 1 | 1 | | | | | | | | | | 5 | | | | | | | | | | | | 2 | | | 1 | 7 | | | | | | | | | 7 | | 2 | | 2 | 3 | M |
| asn | N | | 37 | 2 | | | | | | | | | | 10 | | 1 | | | 13 | | | | 22 | | | | 145 | | 14 | 84 | | | | | | | 1 | 31 | | | 2 | | | 3 | | | N |
| pro | P | | | | | | | | | | | | | | | | | 245 | | | | | | | | | | 1 | 12 | | | | | | | 299 | | | | | | | | | | | P |
| gln | Q | 4 | 32 | | | 14 | | | | | | | | 1 | | | | | 39 | | | 38 | 1 | | | | 2 | | 10 | 3 | | | | | 294 | | 1 | | | | 266 | | | 4 | | | Q |
| arg | R | 48 | 16 | | 2 | 2 | | | | | | | | | | | | 5 | 5 | | | 54 | 21 | | 1 | | 2 | | | | | | | | 1 | | 1 | | | | | | | 2 | | | R |
| ser | S | 1 | 3 | | 1 | 3 | 1 | | | | | | | | 7 | | | 1 | 19 | | 2 | 17 | 8 | | | | 3 | 1 | 1 | 32 | | | | 286 | | 1 | 4 | 21 | | | 4 | | | 8 | | 2 | S |
| thr | T | | 5 | | | 125 | | | | | | 1 | | | | | | 1 | 10 | | | 1 | 3 | | | | | 57 | 8 | | | | | 1 | 1 | | | 207 | | | | | | | | 46 | T |
| val | V | | 4 | 1 | | 80 | 16 | 4 | 1 | 65 | | 3 | | | | | | 34 | 65 | | 1 | | 1 | 1 | | 286 | | 68 | | | | 4 | 108 | | | | | | | | 1 | | | | 1 | 39 | V |
| trp | W | | | | | | | | | | | | | | | | | | | 2 | | | | | | | | | | | | 7 | | | | | | | | | | | | | | | W |
| tyr | Y | | | | | 1 | | | 22 | | | | | | | | | | 4 | 298 | | 1 | | | | | | | | | | 15 | | | | | | 12 | | | | | | 1 | | | Y |
| gap | – | | | 1 | | | | | | | | | | | | | 1 | | | | | | | | | | 1 | | | | | | | | | | | | | | | | | | | | – |

| | | 130 | | | | | 135 | | | | | 140 | | | | | 145 | | | | | 150 | | | | | 155 | | | | | 160 | | | | | 165 | | | | | 170 | | | | | 175 | | | | 179 | |
|---|---|---|---|---|---|---|---|---|---|---|---|---|---|---|---|---|---|---|---|---|---|---|---|---|---|---|---|---|---|---|---|---|---|---|---|---|---|---|---|---|---|---|---|---|---|---|---|---|---|---|---|---|---|
| | | | | | Variable 4 | | | | | | | | | | | | | | | | Motif 4a | | | | | | | | | | | | | | | | | | | | | Motif 4b | | | | | | | | | | | |
| Major | | . | . | l | . | r | s | g | a | . | d | . | . | v | . | D | S | V | a | A | l | . | p | . | a | e | i | e | g | - | e | m | g | d | . | . | . | g | l | q | A | r | l | m | s | q | a | l | r | k | l | . | |
| Ecoli | | D | A | L | A | R | S | G | A | V | D | V | I | V | V | D | S | V | A | A | L | T | P | K | A | E | I | E | G | - | E | I | G | D | S | H | M | G | L | A | A | R | M | M | S | Q | A | M | R | K | L | A | |
| ala | A | | 98 | | 34 | 2 | | 10 | 242 | | 1 | | | | | | | | 296 | 300 | | 1 | | 1 | 265 | | | 2 | 1 | | 2 | | | 4 | 43 | 4 | | 3 | 4 | 22 | 300 | | | | | 1 | 289 | | | | | 32 | A |
| cys | C | 1 | | | | 1 | | | | | | | | | | | | | | | | | | | | | | | | | | | | | | | | | | | | | | | | | | | | | | | C |
| asp | D | 193 | | | | 12 | | | | | 292 | | | | | 300 | | | | | | | | | 2 | 1 | | 50 | 1 | | 73 | | 1 | 289 | | | | | | | | | | | | | | | | | | | D |
| glu | E | 92 | 4 | | | 1 | | 1 | | | | | | | | | | | | | | | | 4 | | 299 | | 240 | | | 217 | | 5 | 6 | | | | | | | | | | | | 1 | | | | 2 | | | E |
| phe | F | | | 3 | 1 | | | | | 1 | | | | | | | | | | | | | | | | | | | | | | | | | | 2 | | | | | | | | | | | | | | | 1 | | F |
| gly | G | 11 | | | | | | 239 | 17 | | 1 | | | | | | | | | | | | | | 1 | | | | 297 | | | | 285 | | | | | 296 | 1 | | | | | | | | 9 | | | | | 3 | G |
| his | H | | 3 | | | | | | | | | | | | | | | | | | | | | | | | | | | | | | | | 2 | 206 | | | | 13 | | | | | | 8 | | | 1 | | | | H |
| ile | I | | 5 | 11 | 66 | | | | | 88 | | 94 | 168 | 32 | 139 | | | | | | | 3 | | | | | 245 | | | | | 48 | | | | | 9 | | 1 | 1 | | | | | | | | | | 2 | 50 | | I |
| lys | K | 1 | 14 | | | 10 | 1 | | 2 | | | | | | | | | | | | | | | 161 | 3 | | | | | | 1 | | 2 | | 1 | 5 | | | 2 | | | 1 | | | | 18 | | | 1 | 290 | | 3 | K |
| leu | L | | 12 | 286 | | | | | 2 | 34 | | 79 | 50 | | | | | | | | 299 | 1 | | 1 | 1 | | 54 | | | | | | | | 3 | 40 | 1 | | 274 | | | | 240 | 9 | | | | 239 | | | 215 | | L |
| met | M | | 73 | | | | | 1 | 1 | | | 26 | 3 | | | | | | | | 1 | | | | | | | | | | | 249 | | | 12 | 3 | 61 | | 3 | | | | 60 | 290 | 1 | | | 61 | | | 31 | | M |
| asn | N | 2 | | | | 4 | | 30 | | | 3 | | | | | | | | | | | | | | 2 | | | 4 | | | 3 | | 1 | | 6 | 2 | | | | | | | | | 1 | | | | | 1 | | 3 | N |
| pro | P | | | | | 1 | 1 | | | | | | | | | | | | | | | | 299 | | | | | | | | | | | | 1 | | 43 | | | | | | | | | | | | | | | | P |
| gln | Q | | 17 | | | | | | | | | | | | | | | | | | | | | 8 | 3 | | | 4 | | | | | | | 37 | 19 | | | 1 | 264 | | | | | | 271 | 1 | | | 1 | 1 | 5 | Q |
| arg | R | | | | | 245 | | | 1 | | | | | | | | | | | | | | | 124 | 1 | | | | | | | | | | | | | 1 | | | | 299 | | | | 1 | | | 296 | 3 | | | R |
| ser | S | | 12 | | 4 | 22 | 292 | 12 | 33 | | 3 | | | | | | 300 | | 1 | | | | | | 18 | | | | 1 | 1 | 2 | | 5 | | 176 | 7 | 1 | | 10 | | | | | | 298 | | 1 | | 2 | | | 54 | S |
| thr | T | | 58 | | 19 | 2 | 6 | 6 | | | | | | | | | | | 3 | | | 106 | 1 | | 2 | | | | | | 1 | | | | 11 | 5 | | | 2 | | | | | | | | | | | | | 199 | T |
| val | V | | 2 | | 176 | | | 1 | 2 | 177 | | 101 | 79 | 268 | 161 | | | 300 | | | | 189 | | | 2 | | | | | | | 2 | | 1 | 8 | 4 | 185 | | 2 | | | | | | | | | | | 1 | 2 | 1 | V |
| trp | W | | | | | | | | | | | | | | | | | | | | | | | | | | | | | | | | | | | | | | | | | | | 1 | | | | | | | | | W |
| tyr | Y | | 2 | | | | | | | | | | | | | | | | | | | | | | | | 1 | | | | | | | | | 3 | | | | | | | | | | | | | | | | | Y |
| gap | – | | | | | | | | | | | | | | | | | | | | | | | | | | | | | 299 | 1 | 1 | 1 | | | | | | | | | | | | | | | | | | | | – |

| % | | sequences | |
|---|---|---|---|
| 0 | | 0 | |
| 10 | | 30 | |
| 25 | | 75 | |
| 50 | 69 | 150 | 209 |
| 70 | 89 | 210 | 269 |
| 90 | 100 | 270 | 300 |

© Roca 2008

Additional File 2

| | | 180 | | | | | 185 | | | | | 190 | | | | | 195 | | | | | 200 | | | | | |
|---|---|---|---|---|---|---|---|---|---|---|---|---|---|---|---|---|---|---|---|---|---|---|---|---|---|---|---|
| | | | | | Variable 5 | | | | | | | | | | | | | Motif 5a | | | | | | | | | |
| Major | | g | . | . | . | . | . | . | t | . | . | i | f | i | N | Q | . | r | . | k | i | g | v | m | - | f | g |
| Ecoli | | G | N | L | K | Q | S | N | T | L | L | I | F | I | N | Q | I | R | M | K | I | G | V | M | - | F | G |
| ala | A | 61 | 65 | 1 | 16 | 2 | 27 | | 4 | 15 | 111 | | | | | | | | | | | | 2 | | | | |
| cys | C | | | | | | | | 76 | 21 | 18 | | | | | | | | | | | | | | | | |
| asp | D | | | | | 1 | | 1 | | | | | | | | | | | 11 | | | | | | | | |
| glu | E | | | | | 1 | | | | | | | | | | | | | 125 | | | | | | | | |
| phe | F | | | | 2 | | | | | | | | 298 | | | | | | | | | | | 8 | | 246 | |
| gly | G | 216 | 2 | | 16 | | | 48 | | | | | | | | | | 1 | 2 | | | 298 | 1 | | 15 | | 299 |
| his | H | 1 | 5 | | 17 | | 2 | 1 | | | | | | | | | | | 1 | | | | | | | | |
| ile | I | | 17 | 172 | | | | | | 60 | 6 | 261 | | 284 | | | 196 | | | | 221 | | 14 | 4 | | | |
| lys | K | | 1 | | 80 | 150 | | 82 | | | | | | | | | | | | 295 | | | | | | | |
| leu | L | | 5 | 101 | | | | | | 73 | 40 | 11 | 1 | 13 | | | 93 | | 7 | | | | 1 | 2 | | 1 | |
| met | M | | | 2 | | | 14 | | | 35 | 3 | 3 | | | | | 1 | | 142 | | 1 | | 11 | 259 | 6 | | |
| asn | N | | 76 | | 110 | 53 | 1 | 150 | | | | | | | 300 | | | | | | | 1 | | | | | |
| pro | P | 3 | | | 2 | | | | | | | | | | | | | | | | 2 | | | | | 6 | |
| gln | Q | | 3 | | 1 | 28 | | 7 | | | | | | | | 300 | | | 9 | | | | | | | | |
| arg | R | | | | | 64 | | 8 | | | | | | | | | | 299 | | 2 | | | | | | | |
| ser | S | 16 | 66 | | 51 | 1 | 170 | 3 | 4 | 2 | | | | | | | | | 2 | | | 1 | | 8 | 1 | | |
| thr | T | 1 | 41 | 1 | | | 84 | | 214 | 81 | 2 | | | 3 | | | 1 | | | 3 | | | 6 | 15 | | | |
| val | V | | 19 | 23 | | | | | 2 | 13 | 120 | 25 | | | | | 9 | | 1 | | 76 | | 265 | 4 | | | |
| trp | W | 1 | | | | | | | | | | | | | | | | | | | | | | | | | |
| tyr | Y | | | | 5 | | 2 | | | | | | 1 | | | | | | | | | | | | | 47 | |
| gap | – | | | | | | | | | | | | | | | | | | | | | | | | 278 | | 1 |

| | | | 205 | | | | | 210 | | | | | 215 | | | | | 220 | | | | | 225 | | | | 229 | |
|---|---|---|---|---|---|---|---|---|---|---|---|---|---|---|---|---|---|---|---|---|---|---|---|---|---|---|---|---|
| | | | | | | | | | | | | Motif 5b | | | | | | | | | | | | | | | | |
| Major | | - | . | p | e | t | t | t | G | g | . | a | l | k | F | y | a | s | v | r | l | d | . | r | r | . | . | |
| Ecoli | | - | N | P | E | T | T | T | G | G | N | A | L | K | F | Y | A | S | V | R | L | D | I | R | R | I | G | |
| ala | A | | | | | | | | | 1 | | 299 | | | | | 214 | | | 1 | | | 2 | | | 48 | 19 | A |
| cys | C | | | | | | | | | | | | | | | | 4 | | 1 | | 3 | | | | | | | C |
| asp | D | | 1 | | | | | | | | | | | | | | | 3 | | | | 234 | | | | | 7 | D |
| glu | E | | | 297 | | | | | | | | | | | | | | | 1 | | | 66 | | | | | 95 | E |
| phe | F | | | | | | | | | | | | | | 300 | 9 | | | | | | | | | | | | F |
| gly | G | 1 | | 1 | | | | | 300 | 299 | | 1 | | | | | | | | | | | | | 16 | 3 | 137 | G |
| his | H | | | | | | | | | | | | | | | | | | | | | | | | 1 | | | H |
| ile | I | | | | | 13 | | | | | 1 | | 1 | | | | | | 30 | | 24 | | 174 | | | 144 | | I |
| lys | K | | | 1 | | | | | | | 39 | | | 299 | | | | | | | | | | 8 | 40 | | | K |
| leu | L | 1 | | | | | | | | | 3 | | 299 | | | | | | 6 | | 239 | | 2 | | | 2 | | L |
| met | M | | | | | 1 | | | | | 1 | | | | | | | | 7 | | 33 | | | | | 2 | | M |
| asn | N | | 179 | 3 | 1 | | | | | | 156 | | | | | | | | | | | | | | | 8 | 1 | N |
| pro | P | | 3 | 293 | | | 1 | 80 | | | | | | | | | | | | | | | | | | | | P |
| gln | Q | 6 | | | | | | | | | 2 | | | | | | | | 2 | | | | | | | | 20 | Q |
| arg | R | | | | | | | | | | 94 | | | 1 | | | | | | 299 | | | | 291 | 241 | | | R |
| ser | S | | 109 | | | 1 | | 2 | | | 1 | | | | | | 79 | 257 | | | | | | 1 | 1 | 18 | 2 | S |
| thr | T | | 8 | 2 | 2 | 270 | 299 | 218 | | | 2 | | | | | | 3 | 39 | 1 | | | | 1 | | | 53 | 18 | T |
| val | V | | | | | 15 | | | | | 1 | | | | | | | | 252 | | 1 | | 121 | | | 22 | | V |
| trp | W | | | | | | | | | | | | | | | | | | | | | | | | | | | W |
| tyr | Y | | | | | | | | | | | | | | | 291 | | 1 | | | | | | | | | | Y |
| gap | – | 292 | | | | | | | | | | | | | | | | | | | | | | | | | 1 | – |

| | | 230 | | | | | | 235 | | | | | | | | 240 | | | | | 245 | | | |
|---|---|---|---|---|---|---|---|---|---|---|---|---|---|---|---|---|---|---|---|---|---|---|---|---|
| | | | | | | | | Variable 6 | | | | | | | | | | | | | | | | |
| Major | | . | . | k | . | - | g | . | . | - | - | - | . | . | g | . | . | t | . | . | k | v | v | K |
| Ecoli | | A | V | K | E | - | G | E | N | - | - | - | V | V | G | S | E | T | R | V | K | V | V | K |
| ala | A | 73 | | | 1 | | 2 | 1 | 1 | 1 | | 1 | 49 | | 1 | 1 | 3 | 16 | 1 | 11 | | | 21 | |
| cys | C | | | | | | | | | | | | | 1 | | | | | | 1 | | | | |
| asp | D | 3 | | | 73 | 2 | 4 | 114 | 74 | 1 | 13 | 2 | 6 | | | | 2 | 1 | | | | | | |
| glu | E | 1 | | 2 | 65 | 1 | 4 | 57 | 142 | | 1 | 2 | 13 | | | | 91 | | 1 | | | | | |
| phe | F | | | | | | 1 | 1 | 4 | | | | 1 | 5 | | | | | | | | | | |
| gly | G | 2 | | 1 | 20 | 7 | 219 | 5 | 1 | 2 | | | | | 298 | 1 | | | | | | | | |
| his | H | 1 | | | | | | | 2 | | | 2 | 6 | | | 2 | 1 | | | | 1 | | | |
| ile | I | 1 | 133 | 4 | | | | 2 | 1 | | 1 | | 60 | 101 | | 15 | 2 | 14 | 2 | 72 | | 27 | 7 | |
| lys | K | 3 | | 282 | 51 | 1 | 12 | 2 | 5 | 12 | 2 | | 1 | 2 | | 17 | 35 | | 124 | | 288 | | 1 | 300 |
| leu | L | 4 | 112 | 3 | 3 | 1 | | | | | | | 1 | 10 | | | 2 | | | 7 | | | 1 | |
| met | M | | 1 | 1 | | | | | 2 | | | | 4 | 11 | | | | | | | 1 | | | |
| asn | N | 5 | | | 17 | 1 | 27 | 24 | 36 | 2 | | 2 | 7 | | | 201 | 2 | | 3 | | 1 | | | |
| pro | P | 3 | 5 | | | 1 | | | | | | | 12 | | | | | | | | | | | |
| gln | Q | 67 | | | 53 | | | 13 | 16 | | | 2 | 4 | | | 1 | 31 | | | | | | | |
| arg | R | | | | 6 | 1 | 23 | | 1 | | | | | | 1 | | 125 | | 167 | | 5 | | | |
| ser | S | 58 | 1 | 2 | 9 | 6 | 6 | 9 | 2 | 1 | | 5 | 6 | 2 | | 54 | | 4 | 1 | 1 | | | 3 | |
| thr | T | 75 | 1 | 4 | 2 | 12 | | 68 | 4 | | | 4 | 4 | 12 | | 3 | 4 | 222 | 1 | | 4 | 4 | 5 | |
| val | V | 3 | 47 | 1 | | 4 | | 1 | 7 | | | | 121 | 146 | | 3 | 2 | 43 | | 208 | | 269 | 262 | |
| trp | W | | | | | | | | | | | | | 2 | | | | | | | | | | |
| tyr | Y | | | | | | | 1 | | | | | 2 | 8 | | 2 | | | | | | | | |
| gap | – | 1 | | | | 263 | 2 | 2 | 2 | 281 | 283 | 280 | 3 | | | | | | | | | | | |

| | | | | 250 | | | | | 255 | | | | | 260 | | | | | 265 | | | | | 269 | |
|---|---|---|---|---|---|---|---|---|---|---|---|---|---|---|---|---|---|---|---|---|---|---|---|---|---|
| | | | | Motif 6a | | | | | | | | | | | | | | Motif 6b | | | | | | | |
| Major | | n | - | k | v | a | p | p | f | . | . | a | e | . | d | i | . | y | g | - | . | g | i | s | |
| Ecoli | | N | - | K | I | A | A | P | F | K | Q | A | E | F | Q | I | L | Y | G | - | E | G | I | N | |
| ala | A | | | | | 249 | 36 | | | | 1 | 239 | 2 | | 2 | | | 1 | 2 | | 5 | 1 | | 3 | A |
| cys | C | | | | 8 | 1 | | | | | | 4 | | | | | | | | | | | | 3 | C |
| asp | D | | | | | | | | | | | | 10 | | 212 | | | | 3 | | 1 | | | 12 | D |
| glu | E | | | | | | | | | | 62 | | 235 | | 34 | | | | | | 163 | | | | E |
| phe | F | | | | | | | | 292 | | | | 9 | 206 | | 1 | 3 | 61 | | | 3 | | 16 | | F |
| gly | G | | | | | | | | | 2 | | 1 | | | | | | | 268 | | | 299 | | | G |
| his | H | | | | | | | | | | | | 1 | | | | | 2 | | 1 | 2 | | | 1 | H |
| ile | I | | | | 24 | | 2 | | | | 37 | 1 | 14 | 7 | 2 | 269 | 23 | | | | | | 254 | | I |
| lys | K | | 1 | 299 | | | 1 | | | 171 | 12 | | 1 | | | | | | 1 | | 50 | | | | K |
| leu | L | | | | 38 | 1 | | | 2 | | 1 | | 3 | 10 | | 9 | 122 | | | | 4 | | 4 | | L |
| met | M | | | | 18 | | | | 1 | | 2 | | 1 | 1 | | 4 | 136 | | | | | | | | M |
| asn | N | 298 | | | | | | | | | 3 | | 6 | | | 1 | | | 23 | | 4 | | | 33 | N |
| pro | P | | | | | 1 | 256 | 299 | | | 1 | 1 | | | | | | | | | | | | | P |
| gln | Q | | | | | | | | | 5 | 124 | | 4 | | 44 | | | | | | 36 | | | | Q |
| arg | R | | | | | | 2 | 1 | | 118 | 4 | | | | | | | | | | 15 | | | | R |
| ser | S | 2 | | 1 | | 48 | 2 | | | | 2 | 2 | 2 | | 2 | | | | 3 | | 10 | | | 236 | S |
| thr | T | | | | 2 | | 1 | | | 4 | 22 | 4 | 3 | 19 | 1 | | 2 | | | | 3 | | 1 | | T |
| val | V | | | | 210 | | | | | | 29 | 48 | 8 | 57 | 1 | 16 | 4 | | | | 4 | | 25 | 2 | V |
| trp | W | | | | | | | | | | | | | | | | | | | | | | | | W |
| tyr | Y | | | | | | | | 4 | | | | 1 | | 2 | | 10 | 236 | | | | | | 10 | Y |
| gap | – | | 299 | | | | | | | | | | | | | | | | | 299 | | | | | – |

| % | | sequences | |
|---|---|---|---|
| 0 | | 0 | |
| 10 | | 30 | |
| 25 | | 75 | |
| 50 | 69 | 150 | 209 |
| 70 | 89 | 210 | 269 |
| 90 | 100 | 270 | 300 |

© Roca 2008

| | | 270 | 271 | 272 | 273 | 274 | 275 | 276 | 277 | 278 | 279 | 280 | 281 | 282 | 283 | 284 | 285 | 286 | 287 |
|---|---|---|---|---|---|---|---|---|---|---|---|---|---|---|---|---|---|---|---|
| | | | | | | | Variable 7 | | | | | | | | | | | | |
| Major | | . | . | g | e | . | . | d | . | . | v | . | . | . | . | . | . | k | . |
| Ecoli | | F | Y | G | E | L | V | D | L | G | V | K | E | K | L | I | E | K | A |
| ala | A | 1 | 9 | 6 | | 2 | 1 | | 6 | 114 | 21 | 12 | 51 | 1 | | | 3 | | 83 |
| cys | C | | | 2 | 20 | | | | | | 1 | | 3 | | | | | | |
| asp | D | | | | 16 | | | 260 | | | | 48 | 1 | 88 | | | 66 | | |
| glu | E | 2 | 145 | | 211 | | | 13 | | | 15 | 95 | 15 | 14 | | | 98 | | |
| phe | F | 8 | 4 | | | | 3 | | 5 | | | | 3 | | 33 | 1 | | | |
| gly | G | 1 | | 263 | 9 | | | 1 | | 181 | | 1 | | 107 | | | 1 | 1 | |
| his | H | 3 | 3 | 2 | | | | 1 | | | | | 46 | | | | 2 | | |
| ile | I | 7 | 8 | 1 | | 99 | 158 | 2 | 24 | | 7 | | 2 | | 151 | 148 | 4 | | |
| lys | K | 65 | | 1 | | | | 12 | | | | 90 | 21 | 58 | | | 30 | 280 | 23 |
| leu | L | 5 | 23 | | | 178 | 70 | | 166 | 2 | 6 | 5 | 61 | | 57 | 9 | 2 | | 1 |
| met | M | | 10 | 1 | | 5 | | | 76 | | | | 6 | | 7 | | 1 | | |
| asn | N | 1 | 14 | 4 | 4 | | | 8 | | | | 11 | 16 | 31 | | | 15 | | 2 |
| pro | P | 5 | | | | | | | | | | | | | | | | | |
| gln | Q | 29 | 13 | | | | | | | | 2 | 27 | 33 | 1 | | | 34 | 1 | |
| arg | R | 143 | 3 | | | | | | | | | 2 | 3 | | | | 31 | 18 | 3 |
| ser | S | 10 | 5 | 18 | 40 | | | | 1 | 3 | 22 | 6 | 1 | | | | 1 | | 186 |
| thr | T | 14 | 35 | | | 1 | | 3 | 2 | | 12 | 2 | 10 | | 1 | | 8 | | 2 |
| val | V | 1 | 19 | | | 15 | 68 | | 6 | | 214 | 1 | 15 | | 51 | 142 | 4 | | |
| trp | W | 3 | | | | | | | | | | | | | | | | | |
| tyr | Y | 2 | 9 | 2 | | | | | 14 | | | | 13 | | | | | | |
| gap | – | | | | | | | | | | | | | | | | | | |

| | 288 | 289 | 290 | 291 | 292 | 293 | 294 | 295 | 296 | 297 | 298 | 299 | 300 | 301 | 302 | 303 | 304 | |
|---|---|---|---|---|---|---|---|---|---|---|---|---|---|---|---|---|---|---|
| | | | | | Motif 7 | | | | | | | | | | | | | |
| Major | g | . | w | . | s | y | . | . | . | . | . | g | q | g | . | . | n | |
| Ecoli | G | A | W | Y | S | Y | K | G | E | K | I | G | Q | G | K | A | N | |
| ala | | 200 | 1 | | 14 | | | 5 | 3 | | | 14 | | | 2 | 44 | 14 | A |
| cys | | | | | | 1 | | | | | | | | | | | | C |
| asp | 1 | | | | | | 16 | 53 | 61 | 1 | | | | | | 52 | | D |
| glu | | 1 | | | | | 42 | 40 | 135 | | | 2 | 3 | 2 | 1 | 187 | | E |
| phe | | | 3 | 106 | | 11 | | | | | | | | | | 1 | 2 | F |
| gly | 299 | | | | 5 | | 43 | 156 | | | 2 | 281 | | 296 | 1 | | 3 | G |
| his | | | 1 | | | 4 | | | 2 | | | | | | | | 1 | H |
| ile | | | | 1 | 1 | 2 | | | 6 | | 180 | | 1 | | 2 | | | I |
| lys | | | | | 1 | | 52 | 3 | 12 | 124 | | 1 | | | 139 | 3 | 20 | K |
| leu | | | | 7 | | 3 | | | 4 | | 114 | | | | 1 | 2 | 1 | L |
| met | | 1 | | | | 1 | | | | | 3 | | | | | | 1 | M |
| asn | | 2 | | | 8 | | 109 | 12 | 14 | 14 | | | | | | 5 | 240 | N |
| pro | | | | | | | | | | | | | | | | 1 | | P |
| gln | | | | | | | 24 | 2 | 27 | 39 | | | 294 | | | 1 | 5 | Q |
| arg | | | | | | | 4 | | 4 | 117 | | | 1 | | 140 | | 2 | R |
| ser | | 93 | 1 | | 228 | | 7 | 24 | 15 | 3 | | 2 | | | 14 | 1 | 5 | S |
| thr | | 1 | | | 35 | | 2 | 5 | 12 | 1 | 1 | | | | | 2 | | T |
| val | | 2 | | | 2 | | | | 5 | | | | 1 | 2 | | | | V |
| trp | | | 292 | | | | | | | | | | | | | | | W |
| tyr | | | 2 | 186 | 6 | 278 | 1 | | | 1 | | | | | | | | Y |
| gap | | | | | | | | | | | | | | | | 1 | 6 | – |

| | | 305 | 306 | 307 | 308 | 309 | 310 | 311 | 312 | 313 | 314 | 315 | 316 | 317 | 318 | 319 | 320 | 321 | 322 | 323 | 324 | 325 | 326 | 327 | 328 |
|---|---|---|---|---|---|---|---|---|---|---|---|---|---|---|---|---|---|---|---|---|---|---|---|---|---|
| | | | | | | | | | | Variable 8a | | | | | | | | | | | | | | | |
| Major | | . | . | . | . | l | . | . | . | . | . | . | . | . | e | . | . | . | . | . | . | . | . | . | . |
| Ecoli | | A | T | A | W | L | K | D | N | P | E | T | A | K | E | I | E | K | K | V | R | E | L | L | L |
| ala | A | 195 | 15 | 28 | 3 | | 35 | 9 | | 11 | 39 | 6 | 143 | 55 | 11 | | 9 | 41 | 22 | | | 32 | 48 | 25 | 14 |
| cys | C | 2 | 14 | | 2 | | 1 | | | | | | 4 | | | | | | | | 1 | | | | |
| asp | D | | 1 | 2 | | | 5 | 66 | 10 | 3 | 73 | | 1 | 58 | 7 | | 44 | 8 | 5 | | | 38 | 1 | 1 | 9 |
| glu | E | | | 42 | 10 | 1 | 25 | 174 | 2 | 8 | 129 | 1 | 3 | 38 | 212 | 1 | 157 | 14 | 12 | 2 | | 124 | 15 | 2 | 11 |
| phe | F | | 1 | | 115 | 7 | | | | 2 | 1 | 6 | 27 | 2 | | | 1 | 5 | 4 | | 7 | | 2 | 20 | 4 |
| gly | G | 1 | 3 | 7 | | | 1 | 1 | | 3 | 1 | | 7 | 3 | | | 1 | 7 | 1 | 1 | | 3 | | 1 | 135 |
| his | H | | | | 2 | | | 1 | 77 | 1 | 2 | | | 6 | | | 1 | 8 | | 1 | | 2 | 19 | 10 | 2 |
| ile | I | | 19 | 13 | | 5 | 1 | 1 | | 3 | | 96 | 2 | 6 | 1 | 204 | 3 | 3 | 4 | 129 | 9 | 1 | 7 | 28 | 4 |
| lys | K | | 127 | 53 | 1 | | 129 | 7 | 5 | 27 | 7 | 3 | 7 | 17 | 17 | 1 | 8 | 77 | 124 | | 29 | 9 | 67 | 1 | 6 |
| leu | L | 7 | 2 | 12 | 12 | 259 | 16 | | | 2 | 3 | 72 | 13 | 9 | 1 | 55 | 2 | 13 | 22 | 50 | 11 | 2 | 23 | 103 | 49 |
| met | M | | 5 | 1 | 2 | 17 | 5 | | | 1 | | 36 | 20 | 3 | 3 | | | | 4 | | 3 | | 18 | 3 | 1 |
| asn | N | | | 45 | | 2 | 3 | 11 | 180 | 3 | 2 | 1 | | 39 | 3 | | 4 | 27 | 1 | 1 | | 12 | 26 | 9 | 7 |
| pro | P | | | | | | | | | 196 | 2 | | | | 2 | 1 | | | 1 | | 1 | | | | |
| gln | Q | 3 | 1 | 44 | 1 | | 3 | 5 | 2 | 3 | 16 | 1 | 2 | 12 | 8 | 1 | 4 | 32 | 28 | 1 | 7 | 26 | 11 | 3 | 11 |
| arg | R | | 86 | 9 | 1 | | 51 | 5 | 8 | 3 | | 2 | 18 | 19 | 5 | | 12 | 19 | 48 | | 202 | 4 | 7 | 3 | 5 |
| ser | S | 16 | 3 | 8 | 3 | | 6 | 2 | 1 | 5 | 7 | | 12 | 7 | 1 | | 22 | 5 | 3 | | | 6 | 29 | 15 | 10 |
| thr | T | 13 | 11 | 25 | 1 | | 2 | 4 | 4 | 15 | 5 | 12 | 14 | 10 | 14 | | 18 | 26 | 7 | 4 | 1 | 7 | 1 | 2 | 7 |
| val | V | 57 | 5 | 4 | 4 | | 6 | 3 | | 3 | 1 | 51 | 8 | 2 | 3 | 25 | 1 | 3 | 1 | 98 | 2 | 13 | 5 | 7 | 2 |
| trp | W | | | | 23 | | | | | | | | | | | | | | | | | | | 1 | |
| tyr | Y | | 1 | | 113 | 1 | | | | | | 1 | 7 | 2 | | | 1 | | | | 7 | | | 45 | 1 |
| gap | – | 6 | 6 | 7 | 7 | 8 | 11 | 11 | 11 | 11 | 12 | 12 | 12 | 12 | 12 | 12 | 12 | 12 | 13 | 13 | 20 | 21 | 21 | 21 | 22 |

| | 329 | 330 | 331 | 332 | 333 | 334 | 335 | 336 | 337 | 338 | 339 | 340 | 341 | 342 | 343 | 344 | 345 | 346 | 347 | 348 | 349 | 350 | 351 | 352 | |
|---|---|---|---|---|---|---|---|---|---|---|---|---|---|---|---|---|---|---|---|---|---|---|---|---|---|
| | | | | | | Variable 8b | | | | | | | | | | | | | | | | | | | |
| Major | . | . | . | . | . | . | . | . | . | . | . | . | . | . | . | . | . | . | . | . | . | . | . | . | |
| Ecoli | S | N | P | N | S | T | P | D | F | S | V | D | D | S | E | G | V | A | E | T | N | E | D | F | |
| ala | 14 | 14 | 59 | 20 | 32 | 30 | 43 | 49 | 47 | 48 | 24 | 29 | 27 | 38 | 33 | 28 | 27 | 27 | 22 | 27 | 19 | 23 | 20 | 13 | A |
| cys | | 1 | 1 | | | | | | 2 | | 1 | | | | | | | | | | | | | | C |
| asp | 4 | 27 | 14 | 25 | 23 | 13 | 16 | 27 | 16 | 27 | 40 | 39 | 41 | 25 | 31 | 36 | 34 | 28 | 31 | 42 | 27 | 13 | 19 | 13 | D |
| glu | 12 | 10 | 44 | 42 | 19 | 41 | 40 | 41 | 29 | 46 | 69 | 61 | 70 | 62 | 63 | 55 | 52 | 43 | 52 | 27 | 30 | 42 | 24 | 13 | E |
| phe | 5 | 1 | 5 | 1 | 5 | 19 | 1 | 6 | 8 | 1 | | 2 | 1 | 2 | 4 | 2 | 3 | | 7 | 6 | 7 | 3 | 1 | 10 | F |
| gly | 4 | 55 | 22 | 22 | 24 | 11 | 19 | 8 | 27 | 15 | 22 | 8 | 14 | 17 | 10 | 15 | 8 | 12 | 5 | 4 | 8 | 5 | 2 | 5 | G |
| his | 6 | 6 | 1 | 5 | 2 | | | 2 | | 3 | 1 | 4 | | | 1 | 1 | 1 | 1 | | | | 1 | | | H |
| ile | 55 | 28 | 4 | 5 | 24 | 10 | 7 | 8 | 4 | 3 | 6 | | 2 | 1 | 1 | | 4 | 3 | 2 | 3 | 4 | 4 | 1 | 1 | I |
| lys | 13 | 21 | 18 | 6 | 9 | 11 | 14 | 8 | 14 | 13 | 7 | 12 | 6 | 14 | 8 | 14 | 7 | 6 | 13 | 2 | 6 | 11 | 4 | 4 | K |
| leu | 60 | 11 | 7 | 14 | 5 | 18 | 33 | 11 | 12 | 1 | 8 | 8 | 12 | 9 | 17 | 15 | 12 | 22 | 14 | 13 | 9 | 5 | 6 | 5 | L |
| met | 8 | | 3 | 19 | 4 | 12 | 3 | 1 | | 7 | 6 | | 2 | | 4 | 8 | 1 | 5 | 1 | | 5 | 4 | | | M |
| asn | 15 | 27 | 9 | 25 | 12 | 12 | 3 | 15 | 14 | 5 | 10 | 3 | 5 | 10 | 5 | 7 | 4 | 5 | 5 | 3 | 10 | 1 | 6 | 8 | N |
| pro | 12 | 23 | 21 | 10 | 28 | 4 | 21 | 24 | 18 | 10 | 8 | 26 | 9 | 13 | 9 | 19 | 11 | 13 | 8 | 9 | 6 | 3 | 3 | 6 | P |
| gln | 5 | | 11 | 7 | 10 | 11 | 8 | 5 | 10 | 10 | 1 | 7 | 2 | 16 | 10 | 5 | 5 | | 5 | 3 | | | 2 | 1 | Q |
| arg | 1 | 3 | 6 | 7 | 6 | 6 | 2 | 1 | 3 | 6 | 3 | 8 | 5 | 1 | 4 | 1 | 7 | 3 | 3 | | | | 1 | | R |
| ser | 24 | 21 | 21 | 19 | 26 | 16 | 13 | 23 | 25 | 24 | 19 | 16 | 12 | 20 | 11 | 7 | 18 | 6 | 7 | 9 | 8 | 9 | 2 | 5 | S |
| thr | 9 | 9 | 12 | 13 | 3 | 28 | 16 | 11 | 16 | 15 | 14 | 18 | 21 | 7 | 15 | 2 | 5 | 13 | 7 | 13 | 9 | 5 | 7 | 5 | T |
| val | 30 | 18 | 13 | 21 | 31 | 18 | 22 | 17 | 11 | 21 | 13 | 10 | 14 | 10 | 11 | 11 | 13 | 8 | 9 | 9 | 7 | 7 | 7 | 6 | V |
| trp | | 1 | | | | 1 | | | | | | | | | | | | | | | | | | | W |
| tyr | | | | 5 | | | | | | | | 1 | 3 | | | | 2 | | | | 1 | 1 | | | Y |
| gap | 23 | 24 | 29 | 34 | 37 | 39 | 39 | 43 | 44 | 45 | 48 | 48 | 54 | 55 | 63 | 74 | 86 | 105 | 109 | 130 | 144 | 163 | 195 | 205 | – |

| % | | sequences |
|---|---|---|
| 0 | | 0 |
| 10 | | 30 |
| 25 | | 75 |
| 50 | 69 | 150 209 |
| 70 | 89 | 210 269 |
| 90 | 100 | 270 300 |

Additional File 2. Detailed ProfileGrid of the RecA protein family. The frequency values were calculated over the full length (352 residues) of the E. coli RecA homolog (top sequence) which determines the position numbering. The "Major" summary line is the 187 residues conserved above a 70% majority threshold. The 12 RecA family motifs are boxed and labeled (as in Additional file 1) while the connecting variable regions are only labeled. Frequency values are shaded in the ranges of 50 to 69% (light gray), 70 to 89% (dark gray), and 90 to 100% (black). Since we anticipate updating the analysis in the future, this is version 1.0 of the RecA ProfileGrid.

© Roca 2008
